# Statistics of spike trains in conductance-based neural networks: Rigorous results

**DOI:** 10.1186/2190-8567-1-8

**Published:** 2011-08-25

**Authors:** Bruno Cessac

**Affiliations:** 1 NeuroMathComp, INRIA, 2004 Route des Lucioles, 06902, Sophia-Antipolis, France

## Abstract

We consider a conductance-based neural network inspired by the generalized Integrate and Fire model introduced by Rudolph and Destexhe in 1996. We show the existence and uniqueness of a unique Gibbs distribution characterizing spike train statistics. The corresponding Gibbs potential is explicitly computed. These results hold in the presence of a time-dependent stimulus and apply therefore to non-stationary dynamics.

## 1 Introduction

 Neural networks have an overwhelming complexity. While an isolated neuron can exhibit a wide variety of responses to stimuli [1], from regular spiking to chaos [2,3], neurons coupled in a network via synapses (electrical or chemical) may show an even wider variety of collective dynamics [4] resulting from the conjunction of non-linear effects, time propagation delays, synaptic noise, synaptic plasticity, and external stimuli [5]. Focusing on the action potentials, this complexity is manifested by drastic changes in the spikes activity, for instance when switching from spontaneous to evoked activity (see for example A. Riehle’s team experiments on the monkey motor cortex [6-9]). However, beyond this, complexity may exist some hidden laws ruling an (hypothetical) “neural code” [10].

 One way of unraveling these hidden laws is to seek some regularities or reproducibility in the statistics of spikes. While early investigations on spiking activities were focusing on firing rates where neurons are considered as independent sources, researchers concentrated more recently on collective statistical indicators such as pairwise correlations. Thorough experiments in the retina [11,12] as well as in the parietal cat cortex [13] suggested that such correlations are crucial for understanding spiking activity. Those conclusions where obtained using the *maximal entropy principle*[14]. Assume that the average value of observables quantities (e.g., firing rate or spike correlations) has been measured. Those average values constitute constraints for the statistical model. In the maximal entropy principle, *assuming stationarity*, one looks for the probability distribution which maximizes the statistical entropy given those constraints. This leads to a (time-translation invariant) Gibbs distribution. In particular, fixing firing rates and the probability of pairwise coincidences of spikes lead to a Gibbs distribution having the same form as the Ising model. This idea has been introduced by Schneidman et al. in [11] for the analysis of retina spike trains. They reproduce accurately the probability of *spatial* spiking pattern. Since then, their approach has known a great success (see, e.g., [15-17]), although some authors raised solid objections on this model [12,18-20] while several papers have pointed out the importance of *temporal* patterns of activity at the network level [21-23]. As a consequence, a few authors [13,24,25] have attempted to define time-dependent models of Gibbs distributions where constraints include time-dependent correlations between pairs, triplets, and so on [26]. As a matter of fact, the analysis of the data of [11] with such models describes more accurately the statistics of *spatio-temporal* spike patterns [27].

Taking into account all constraints inherent to experiments, it seems extremely difficult to find an optimal model describing spike trains statistics. It is in fact likely that there is not one model, but many, depending on the experiment, the stimulus, the investigated part of the nervous system and so on. Additionally, the assumptions made in the works quoted above are difficult to control. Especially, the maximal entropy principle assumes a stationary dynamics while many experiments consider a time-dependent stimulus generating a time-dependent response where the stationary approximation may not be valid. At this stage, having an example where one knows the explicit form of the spike trains, probability distribution would be helpful to control those assumptions and to define related experiments.

This can be done considering neural network models. Although, to be tractable, such models may be quite away from biological plausibility, they can give hints on which statistics can be expected in real neural networks. But, even in the simplest examples, characterizing spike statistics arising from the conjunction of non-linear effects, time propagation delays, synaptic noise, synaptic plasticity, and external stimuli is far from being trivial on mathematical grounds.

 In [28], we have nevertheless proposed an exact and explicit result for the characterization of spike trains statistics in a discrete-time version of Leaky Integrate-and-Fire neural network. The results were quite surprising. It has been shown that whatever the parameters value (in particular synaptic weights), spike trains are distributed according to a Gibbs distribution whose potential can be explicitly computed. The first surprise lies in the fact that this potential has infinite range, namely spike statistics has an infinite memory. This is because the membrane potential evolution integrates its past values and the past influence of the network via the leak term. Although leaky integrate and fire models have a reset mechanism that erases the memory of the neuron whenever it spikes, it is not possible to upper bound the next time of firing. As a consequence, statistics is non-Markovian (for recent examples of non-Markovian behavior in neural models see also [29]). The infinite range of the potential corresponds, in the maximal entropy principle interpretation, to having infinitely many constraints.

 Nevertheless, the leak term influence decays exponentially fast with time (this property guarantees the existence and uniqueness of a Gibbs distribution). As a consequence, one can approximate the exact Gibbs distribution by the invariant probability of a Markov chain, with a memory depth proportional to the log of the (discrete time) leak term. In this way, the truncated potential corresponds to a finite number of constraints in the maximal entropy principle interpretation. However, the second surprise is that this approximated potential is nevertheless far from the Ising model or any of the models discussed above, which appear as quite bad approximations. In particular, there is a need to consider *n*-uplets of spikes with time delays. This mere fact asks hard problems about evidencing such type of potentials in experiments. Especially, new type of algorithms for spike trains analysis has to be developed [30].

 The model considered in [28] is rather academic: time evolution is discrete, synaptic interactions are instantaneous, dynamics is stationary (the stimulus is time-constant) and, as in a leaky integrate and fire model, conductances are constant. It is therefore necessary to investigate whether our conclusions remain for more realistic neural networks models. In the present paper, we consider a conductance-based model introduced by Rudolph and Destexhe in [31] called “generalized Integrate and Fire” (gIF) model. This model allows one to consider realistic synaptic responses and conductances depending on spikes arising in the past of the network, leading to a rather complex dynamics which has been characterized in [32] in the deterministic case (no noise in the dynamics). Moreover, the biological plausibility of this model is well accepted [33,34].

 Here, we analyze spike statistics in the gIF model with noise and with a time-dependent stimulus. Moreover, the post-synaptic potential profiles are quite general and summarize all the examples that we know in the literature. Our main result is to prove the existence and uniqueness of a Gibbs measure characterizing spike trains statistics, for all parameters compatible with physical constraints (finite synaptic weights, bounded stimulus, and positive conductances). Here, as in [28], the corresponding Gibbs potential has infinite range corresponding to a non-Markovian dynamics, although Markovian approximations can be proposed in the gIF model too. The Gibbs potential depends on all parameters in the model (especially connectivity and stimulus) and has a form quite more complex than Ising-like models. As a by-product of the proof of our main result, additional interesting notions and results are produced such as continuity, with respect to a raster, or exponential decay of memory thanks to the shape of synaptic responses.

 The paper is organized as follows. In Section 2, we briefly introduce integrate and fire models and propose two important extensions of the classical models: the spike has a duration and the membrane potential is reset to a non-constant value. These extensions, which are necessary for the validity of our mathematical results, render nevertheless the model more biologically plausible (see Section 9). One of the keys of the present work is to consider spike trains (raster plots) as infinite sequences. Since in gIF models, conductances are updated upon the occurrence of spikes, one has to consider two types of variables with distinct type of dynamics. On the one hand, the membrane potential, which is the physical variable associated with neurons dynamics, evolves continuously. On the other hand, spikes are discrete events. Conductances are updated according to these discrete-time events. The formalism introduced in Sections 2 and 3 allows us to handle properly this mixed dynamics. As a consequence, these sections define gIF model with more mathematical structure than the original paper [31] and mostly contain original results. Moreover, we add to the model several original features such as the consideration of a general form of synaptic profile with exponential decay or the introduction of noise. Section 4 proposes a preliminary analysis of gIF model dynamics. In Sections 5 and 6, we provide several useful mathematical propositions as a necessary step toward the analysis of spike statistics, developed in Section 7, where we prove the main result of the paper: existence and uniqueness of a Gibbs distribution describing spike statistics. Sections 8 and 9 are devoted to a discussion on practical consequences of our results for neuroscience.

## 2 Integrate and fire model

We consider the evolution of a set of *N* neurons. Here, neurons are considered as “points” instead of spatially extended and structured objects. As a consequence, we define, for each neuron k∈{1,…,N}, a variable $V_{k}(t)$ called the “membrane potential of neuron *k* at time *t*” without specification of which part of a real neuron (axon, soma, dendritic spine, …) it corresponds to. Denote V(t) the vector ${\left(V_{k}(t)\right)}_{k=1}^{N}$.

We focus here on “integrate and fire models”, where dynamics always consists of two regimes.

### 2.1 The “integrate regime”

 Fix a real number *θ* called the “firing threshold of the neuron”.^1^ Below the threshold, $V_{k}<\theta$, neuron *k*’s dynamics is driven by an equation of the form:

(1) $$C_{k}\frac{dV_{k}}{dt}+g_{k}V_{k}=i_{k},$$

where $C_{k}$ is the membrane capacity of neuron *k*. In its most general form, the neuron *k*’s membrane conductance $g_{k}>0$ depends on $V_{k}$ plus additional variables such as the probability of having ionic channels open (see, e.g., Hodgkin-Huxley equations [35]) as well as on time *t*. The explicit form of $g_{k}$ in the present model is developed in Section 3.4. The current $i_{k}$ typically depends on time *t* and on the past activity of the network. It also contains a stochastic component modeling noise in the system (e.g., synaptic transmission, see Section 3.5).

### 2.2 LIF model

 A classical example of integrate and fire model is the Leaky Integrate and Fire’s (LIF) introduced in [36] where Equation (1) reads:

(2) $$\frac{dV_{k}}{dt}=-\frac{V_{k}}{\tau_{L}}+\frac{i_{k}(t)}{C_{k}}.$$

where $g_{k}$ is a constant and $\tau_{L}=\frac{C_{k}}{g_{k}}$ is the characteristic time for membrane potential decay when no current is present (“leak term”).

### 2.3 Spikes

**Fig. 1:**
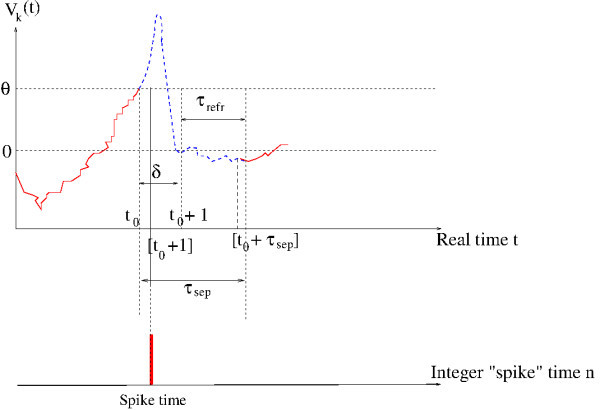
Time course of the membrane potential in our model. The *blue dashed curve* illustrates the shape of a real spike, but what we model is the *red curve*.

The dynamical evolution (1) may eventually lead $V_{k}$ to exceed *θ*. If, at some time *t*, $V_{k}(t)=\theta$, then neuron *k* emits a spike or “fires”. In our model, like in biophysics, a spike has a finite duration δ>0; this is a generalization of the classical formulation of integrate and fire models where the spike is considered instantaneous. On biophysical grounds, *δ* is of order of a millisecond. Changing the time units, we may set δ=1 without loss of generality. Additionally, neurons have a refractory period $\tau_{\mathrm{refr}}>0$ where they are not able to emit a new spike although their membrane potential can fluctuate below the threshold (see Figure 1). Hence, spikes emitted by a given neuron are separated by a minimal time scale

(3) $$\tau_{\mathrm{sep}}=\delta +\tau_{\mathrm{refr}}.$$

### 2.4 Raster plots

 In experiments, spiking neuron activity is represented by “raster plots”, namely a graph with time in abscissa, and a neuron labeling in ordinate such that a vertical bar is drawn each “time” a neuron emits a spike. Since spikes have a finite duration *δ* such a representation limits the time resolution: events with a time scale smaller than *δ* are not distinguished. As a consequence, if neuron 1 fires at time $t_{1}$ and neuron 2 at time $t_{2}$ with $|t_{2}-t_{1}|<\delta =1$ the two spikes appear to be simultaneous on the raster. Thus, the raster representation introduces a time quantization and has a tendency to enhance synchronization. In gIF models, conductances are updated upon the occurrence of spikes (see Section 3.2) which are considered as such discrete events. This could correspond to the following “experiment”. Assume that we measure the spikes emitted by a set of in vitro neurons and that we use this information to update the conductances of a model, in order to see how this model “matches” the real neurons (see [34] for a nice investigation in this spirit). Then, we would have to take into account that the information provided by the experimental raster plot is discrete, even if the membrane potential evolves continuously. The consequences of this time discretization as well as the limit δ→0 are developed in the discussion section.

As a consequence, one has to consider two types of variables with distinct type of dynamics. On the one hand, the membrane potential, which is the physical variable associated with neuron dynamics, evolves with a continuous time. On the other hand, spikes, which are the quantities of interest in the present paper, are discrete events. To properly define this mixed dynamics and study its properties, we have to model spikes times and raster plots.

### 2.5 Spike times

If, at time *t*, $V_{k}(t)=\theta$, a spike is registered at the *integer time immediately aftert*, called the spike time. Choosing integers for the spike time occurrence is a direct consequence of setting δ=1. Thus, to each neuron *k* and integer *n*, we associate a “spiking state” defined by:

$$\omega_{k}(n)=\left\{ \begin{array}{ll} 1 & \text{if }\exists t\in \;]n-1,n]\;such\;that\;V_{k}(t)=\theta ;\\ 0 & \text{otherwise.} \end{array}\right.$$

For convenience and in order to simplify the notations in the mathematical developments, we call [t] the largest integer which is ≤*t* (thus [−1.2]=−2 and [1.2]=1). Thus, the integer immediately after *t* is [t+1] and we have therefore that $\omega_{k}([t+1])=1$ whenever $V_{k}(t)=\theta$. Although, characteristic events in a raster plot are spikes (neuron fires), it is useful in subsequent developments to consider also the case when neuron is not firing ($\omega_{k}(n)=0$).

### 2.6 Reset

 In the classical formulation of integrate and fire models, the spike occurs *simultaneously* with a reset of the membrane potential to some *constant* value $V_{\mathrm{reset}}$, called the “reset potential”. Instantaneous reset is a source of pathologies as discussed in [32,37] and in the discussion section. Here, we consider that reset occurs after the time delay $\tau_{\mathrm{sep}}\geq 1$ including spike duration and refractory period. We set:

(4) $$V_{k}(t)=\theta \Rightarrow V_{k}\left([t+\tau_{\mathrm{sep}}]\right)=V_{\mathrm{reset}}.$$

The reason why the reset time is the integer number $\left[t+\tau_{\mathrm{sep}}\right]$ instead of the real $t+\tau_{\mathrm{sep}}$ is that it eases the notations and proofs. Since the reset value is random (see below and Figure 1), this assumption has no impact on the dynamics.

 Indeed, in our model, the reset value $V_{\mathrm{reset}}$ is not a constant. This is a Gaussian random variable with mean zero (we set the rest potential to zero without loss of generality) and variance $\sigma_{R}^{2}>0$. In this way, we model the spike duration and refractory period, as well as the random oscillations of the membrane potential during the refractory period. As a consequence, the value of $V_{k}$ when the neuron can fire again is not a constant, as it is in classical IF models. A related reference (spiking neurons with partial reset) is [38]. The assumption that $\sigma_{R}^{2}>0$ is necessary for our mathematical developments (see the bounds (37)). We assume $\sigma_{R}^{2}$ to be small to avoid trivial and unrealistic situations where $V_{\mathrm{reset}}\geq \theta$ with a large probability leading the neuron to fire all the time. Note, however, that this is not a required assumption to establish our mathematical results. We also assume that, in successive resets, the random variables $V_{\mathrm{reset}}$ are independent.

### 2.7 The shape of membrane potential during the spike

 On biophysical grounds, the time course of the membrane potential during the spike includes a depolarization and re-polarization phase due to the non-linear effects of gated ionic channels on the conductance. This leads to introduce, in modeling, additional variables such as activation/inactivation probabilities as in the Hodgkin-Huxley model [35] or adaptation current as, e.g., in FitzHugh-Nagumo model [39-42] (see the discussion section for extensions of our results to those models). Here, since we are considering only one variable for the neuron state, the membrane potential, we need to define the spike profile, i.e., the course of $V_{k}(t)$ during the time interval $\left(t,[t+\tau_{\mathrm{sep}}]\right)$. It turns out that the precise shape of this profile plays no role in the developments proposed here, where we concentrate on spike statistics. Indeed, a spike is registered whenever $V_{k}(t)=\theta$, and this does not depend on the spike shape. What we need is therefore to define the membrane potential evolution before the spike, given by (1), and after the spike, given by (4) (see Figure 1).

### 2.8 Mathematical representation of raster plots

The “spiking pattern” of the neural network at integer time *n* is the vector $\omega (n)={\left(\omega_{k}(n)\right)}_{k=1}^{N}$. For m<n, we note $\omega_{m}^{n}=\{\omega (m)\omega (m+1)\cdots \omega (n)\}$ the ordered sequence of spiking patterns between *m* and *n*. Such sequences are called *spike blocks*. Additionally, we note that $\omega_{m}^{n_{1}-1}\omega_{n_{1}}^{n}=\omega_{m}^{n}$, the concatenation of the blocks $\omega_{m}^{n_{1}-1}$ and $\omega_{n_{1}}^{n}$.

Call $A={\left\{0,1\right\}}^{N}$ the set of spiking patterns (alphabet). An element of $X\overset{\mathrm{def}}{=}A^{Z}$, i.e., a bi-infinite ordered sequence $\omega ={\left\{\omega (n)\right\}}_{n=-\infty }^{+\infty }$ of spiking patterns, is called a “raster plot”. It tells us which neurons are firing at each time n∈Z. In experiments, raster plots are obviously finite sequences of spiking pattern but the extension to , especially the possibility of considering an arbitrary distant past (negative times), is a key of the present work. In particular, the notation $\omega_{-\infty }^{n}$ refers to spikes occurring from −∞ to *n*.

To each raster ω∈X and each neuron index j=1,…,N, we associate an ordered (generically infinite) list of “spike times” ${\left\{t_{j}^{\left(r\right)}(\omega )\right\}}_{r=1}^{\infty }$ (integer numbers) such that $t_{j}^{\left(r\right)}(\omega )$ is the *r*-th time of firing of neuron *j* in the raster *ω*. In other words, we have $\omega_{j}(n)=1$ if and only if $n=t_{j}^{\left(r\right)}(\omega )$ for some r=1,…,+∞. We use here the following convention. The index *k* is used for a post-synaptic neuron while the index *j* refers to pre-synaptic neurons. Spiking events are used to update the conductance of neuron *k* according to spikes emitted by pre-synaptic neurons. That is why we label the spike times with an index *j*.

We introduce here two specific rasters which are of use in the paper. We note Ω_0_ the raster such that $\omega_{k}(n)=0$, ∀k=1,…,N, ∀n∈Z (no neuron ever fires) and Ω_1_ the raster $\omega_{k}(n)=1$, ∀i=1,…,N, ∀n∈Z (each neuron is firing at every integer time).

 Finally, we use the following notation borrowed from [43]. We note, for n∈Zm≥0, and *r* integer:

(5) $$\omega \overset{m,n}{=}\omega^{\prime }\;\text{if }\omega (r)=\omega^{\prime }(r),\forall r\in \{n-m,\ldots ,n\}.$$

For simplicity, we consider that $\tau_{\mathrm{ref}}$, the refractory period, is smaller than 1 so that a neuron can fire two consecutive time steps (i.e., one can have $\omega_{k}(n)=1$ and $\omega_{k}(n+1)=1$). This constraint is discussed in Section 9.2.

### 2.9 Representation of time-dependent functions

Throughout the paper, we use the following convention. For a real function of *t* and *ω*, we write f(t,ω) for $f(t,\omega_{-\infty }^{\left[t\right]})$ to simplify notations. This notation takes into account the duality between variables such as membrane potential evolving with respect to a continuous time and raster plots labeled with discrete time. Thus, the function f(t,ω) is a function of the continuous variable *t* and of the spike block $\omega_{-\infty }^{\left[t\right]}$, where by definition [t]≤t, namely f(t,ω) depends on the spike sequences occurring *beforet*. This constraint is imposed by causality.

### 2.10 Last reset time

We define $\tau_{k}(t,\omega )$ as the last time before *t* where neuron *k*’s membrane potential has been reset, in the raster *ω*. This is −∞ if the membrane potential has never been reset. As a consequence of our choice (4) for the reset time $\tau_{k}(t,\omega )$ is an integer number fixed by *t* and the raster before *t*. The membrane potential value of neuron *k* at time *t* is controlled by the reset value $V_{\mathrm{reset}}$ at time $\tau_{k}(t,\omega )$ and by the further subthreshold evolution (1) from time $\tau_{k}(t,\omega )$ to time *t*.

## 3 Generalized integrate and fire models

 In this paper, we concentrate on an extension of (2), called “generalized Integrate-and-Fire” (gIF), introduced in [31], closer to biology [33,34], since it considers more seriously neurons interactions via synaptic responses.

### 3.1 Synaptic conductances

 Depending on the neurotransmitter they use for synaptic transmission (AMPA, NMDA, GABA A, GABA B [44]), neurons can be excitatory (population ) or inhibitory (population ). This is modeled by introducing reversal potentials $E^{+}$ for excitatory (typically $E^{+}≃0\text{ mV}$ for AMPA and NMDA) and $E^{-}$ for inhibitory ($E^{-}≃-70\text{ mV}$ for GABA A and $E^{-}≃-95\text{ mV}$ for GABA B). We focus here on one population of excitatory and one population of inhibitory neurons although extensions to several populations may be considered as well. Also, each neuron is submitted to a current $I_{k}(t)$. We assume that this current has some stochastic component that mimics synaptic noise (Section 3.5).

The variation in the membrane potential of neuron *k* at time *t* reads:

(6) $$C_{k}\frac{dV_{k}}{dt}=-g_{L,k}(V_{k}-E_{L})-g_{k}^{\left(E\right)}(t)\left(V_{k}-E^{+}\right)-g_{k}^{\left(I\right)}(t)\left(V_{k}-E^{-}\right)+I_{k}(t),$$

where $g_{L,k}$ is a leak conductance, $E_{L}<0$ is the leak reversal potential (about −65 mV), $g_{k}^{\left(E\right)}(t)$ the conductance of the excitatory population and $g_{k}^{\left(I\right)}(t)$ the conductance of inhibitory population. They are given by:

(7) $$g_{k}^{\left(E\right)}(t)=\sum_{j\in E}g_{kj}(t);\;g_{k}^{\left(I\right)}(t)=\sum_{j\in I}g_{kj}(t),$$

where $g_{kj}$ is the conductance of the synaptic contact j→k.

We may rewrite Equation (6) in the form (1) setting

$$g_{k}(t)=g_{L,k}+g_{k}^{\left(E\right)}(t)+g_{k}^{\left(I\right)}(t),$$

and

$$i_{k}(t)=g_{L,k}E_{L}+g_{k}^{\left(E\right)}(t)E^{+}+g_{k}^{\left(I\right)}(t)E^{-}+I_{k}(t).$$

### 3.2 Conductance update upon a spike occurrence

The conductances $g_{kj}(t)$ in (7) depend on time *t* but also on pre-synaptic spikes occurring before *t*. This is a general statement, which is modeled in gIF models as follows. Upon arrival of a spike in the pre-synaptic neuron *j* at time $t_{j}^{\left(r\right)}(\omega )$, the membrane conductance of the post-synaptic neuron *k* is modified as:

(8) $$g_{kj}(t)=g_{kj}\left(t_{j}^{\left(r\right)}(\omega )\right)+G_{kj}\alpha_{kj}\left(t-t_{j}^{\left(r\right)}(\omega )\right),\;t>t_{j}^{\left(r\right)}(\omega ).$$

In this equation, the quantity $G_{kj}\geq 0$ characterizes the maximal amplitude of the conductance during a post-synaptic potential. We use the convention that $G_{kj}=0$ if and only if there is no synapse between *j* and *k*. This allows us to encode the graph structure of the neural network in the matrix *G* with entries $G_{kj}$. Note that the $G_{kj}$’s can evolve in time due to synaptic plasticity mechanisms (see Section 9.4).

 The function $\alpha_{kj}$ (called “alpha” profile [44]) mimics the time course of the synaptic conductance upon the occurrence of the spike. Classical examples are:

(9) $$\alpha_{kj}(t)=e^{-\frac{t}{\tau_{kj}}}H(t),$$

(exponential profile) or:

(10) $$\alpha_{kj}(t)=\frac{t}{\tau_{kj}}e^{-\frac{t}{\tau_{kj}}}H(t),$$

with *H* the Heaviside function (that mimics causality) and $\tau_{kj}$ is the characteristic decay times of the synaptic response. Since *t* is a time, the division by $\tau_{kj}$ ensures that $\alpha_{kj}(t)$ is a dimensionless quantity: this eases the legibility of the subsequent equations on physical grounds (dimensionality of physical quantities).

 Contrarily to (9) the synaptic profile (10), with $\alpha_{kj}(0)=0$ while $\alpha_{kj}(t)$ is maximal for $t=\tau_{kj}$, allows one to smoothly delay the spike action on the post-synaptic neuron. More general forms of synaptic responses could be considered as well. For example, the *α* profile may obey a Green equation of type [45]:

$$\sum_{l=0}^{k}a_{kj}^{\left(l\right)}\frac{d^{l}\alpha_{kj}}{du^{l}}(t)=\delta (t),$$

where k=1$a_{kj}^{\left(0\right)}=\frac{1}{\tau_{kj}}$$a_{kj}^{\left(1\right)}=1$, corresponds to (9), and so on.

### 3.3 Mathematical constraints on the synaptic responses

 In all the paper, we assume that the $\alpha_{kj}$’s are positive and bounded. Moreover, we assume that:

(11) $$\alpha_{kj}(t)∼\frac{t^{d}}{\tau_{kj}^{d}}e^{-\frac{t}{\tau_{kj}}}\overset{\mathrm{def}}{=}f_{kj}(t),\;t\to +\infty ,$$

for some integer *d*. So that $\alpha_{kj}(t)$ decays exponentially fast as t→+∞, with a characteristic time $\tau_{kj}$, the decay time of the evoked post-synaptic potential. This constraint matches all synaptic response kernels that we know (where typically d=0,1) [44,45].

This has the following consequence. For all *t*, M<t integer, *r* integer, we have, setting t={t}+[t], where {t} is the fractional part:

$$\sum_{r<M}\alpha_{kj}(t-r)=\sum_{r<M}\alpha_{kj}\left([t]-r+\{t\}\right)=\sum_{n>[t]-M}\alpha_{kj}\left(n+\{t\}\right).$$

Therefore, as M→−∞,

$$\sum_{r<M}\alpha_{kj}(t-r)∼\sum_{n\geq [t]-M+1}f_{kj}\left(n+\{t\}\right)<\int_{t-M}^{+\infty }f_{kj}(u)\;du=P_{d}\left(\frac{t-M}{\tau_{kj}}\right)e^{-\frac{t-M}{\tau_{kj}}},$$

where $P_{d}()$ is a polynomial of degree *d*.

We introduce the following (Hardy) notation: if a function f(t) is bounded from above, as t→+∞, by a function g(t) we write: f(t)⪯g(t). Using this notation, we have therefore:

**Proposition 1**

(12) $$\sum_{r<M}\alpha_{kj}(t-r)⪯P_{d}\left(\frac{t-M}{\tau_{kj}}\right)e^{-\frac{t-M}{\tau_{kj}}},$$

*as*M→−∞.

Additionally, the constraint (11) implies that there is some $\alpha^{+}<+\infty$ such that, for all *t*, for all *k*, *j*:

(13) $$\sum_{r<t}\alpha_{kj}(t-r)\leq \alpha^{+}.$$

Indeed, for n≥0 integer, call $A_{kj}(n)=\sup\{\alpha_{kj}(n+x);x\in [0,1[\}$. Then,

$$\sum_{r<t}\alpha_{kj}(t-r)=\sum_{n=1}^{+\infty }\alpha_{kj}\left(n+\{t\}\right)\leq \sum_{n=1}^{+\infty }A_{kj}(n).$$

Due to (11), this series converges (e.g., from Cauchy criterion). We set:

$$\alpha_{+}=\max_{kj}\sum_{n=1}^{+\infty }A_{kj}(n).$$

On physical grounds, it implies that the conductance $g_{k}$ remains bounded, even if each pre-synaptic neuron is firing all the time (see Equation (29) below).

### 3.4 Synaptic summation

Assume that Equation (8) remains valid for an arbitrary number of pre-synaptic spikes emitted by neuron *j* within a finite time interval [s,t] (i.e., neglecting non-linear effects such as the fact that there is a finite amount of neurotransmitter leading to saturation effects). Then, one obtains the following equation for the conductance $g_{kj}$ at time *t*, upon the arrival of spikes at times $t_{j}^{\left(r\right)}(\omega )$ in the time interval [s,t]:

$$g_{kj}(t)=g_{kj}(s)+G_{kj}\sum_{\left\{r;s\leq t_{j}^{\left(r\right)}(\omega )<t\right\}}\alpha_{kj}\left(t-t_{j}^{\left(r\right)}(\omega )\right).$$

The conductance at time *s*, $g_{kj}(s)$, depends on the neuron *j*’s activity preceding *s*. This term is therefore unknown unless one knows exactly the past evolution before *s*. One way to circumvent this problem is to taking *s* arbitrary far in the past, i.e., taking s→−∞ in order to remove the dependence on initial conditions. This corresponds to the following situation. When one observes a real neural network, the time where the observation starts, say t=0, is usually not the time when the system has begun to exist, *s* in our notations. Taking *s* arbitrary far in the past corresponds to assuming that the system has evolved long enough so that it has reached sort of a “permanent regime”, not necessarily stationary, when the observation starts. On phenomenological grounds, it is enough to take −*s* larger than all characteristic relaxation times in the system (e.g., leak rate and synaptic decay rate). Here, for mathematical purposes, it is easier to take the limit s→−∞.

Since $g_{kj}(t)$ depends on the raster plot up to time *t*, via the spiking times $t_{j}^{\left(r\right)}(\omega )$, this limit makes only sense when taking it “conditionally” to a prescribed raster plot *ω*. In other words, one can know the value of the conductances $g_{kj}$ at time *t* only if the past spike times of the network are known. We write $g_{kj}(t,\omega )$ from now on to make this dependence explicit.

We set

(14) $$\begin{array}{rc} \alpha_{kj}(t,\omega ) & =\lim_{s\to -\infty }\sum_{\left\{r;s\leq t_{j}^{\left(r\right)}(\omega )<t\right\}}\alpha_{kj}\left(t-t_{j}^{\left(r\right)}(\omega )\right)\\ & \equiv \sum_{\left\{r;t_{j}^{\left(r\right)}(\omega )<t\right\}}\alpha_{kj}\left(t-t_{j}^{\left(r\right)}(\omega )\right), \end{array}$$

with the convention that $\sum_{\emptyset }=0$ so that $\alpha_{kj}(t,\Omega_{0})=0$ (recall that Ω_0_ is the raster such that no neuron ever fires). The limit (14) exists from (13).

### 3.5 Noise

We allow, in the definition of the current $I_{k}(t)$ in Equation (6), the possibility of having a stochastic term corresponding to noise so that:

(15) $$I_{k}(t)=i_{k}^{\left(\mathrm{ext}\right)}(t)+\sigma_{B}\xi_{k}(t),$$

where $i_{k}^{\left(\mathrm{ext}\right)}(t)$ is a deterministic external current and $\xi_{k}(t)$ a noise term whose amplitude is controlled by $\sigma_{B}>0$. The model affords an extension where $\sigma_{B}$ depends on *k* but this extension is straightforward and we do not develop it here. The noise term can be interpreted as the random variation in the ionic flux of charges crossing the membrane per unit time at the post-synaptic button, upon opening of ionic channels due to the binding of neurotransmitter.

We assume that $\xi_{k}(t)$ is a white noise, $\xi_{k}(t)=\frac{dB_{k}}{dt}$ where $dB_{k}(t)$ is a Wiener process, so that $dB(t)={\left(dB_{k}(t)\right)}_{k=1}^{N}$ is a *N*-dimensional Wiener process. Call *P* the noise probability distribution and E[] the expectation under *P*. Then, by definition, $E[dB_{k}(t)]=0$, ∀k=1,…,N, t∈R, and $E[dB_{k}(s)\;dB_{l}(t)]=\delta_{kl}\delta (t-s)\;dt$ where $\delta_{kl}=1$ if l=k, l,k=1,…,N and δ(t−s) is the Dirac distribution.

### 3.6 Differential equation for the integrate regime of gIF

Summarizing, we write Equation (6) in the form:

(16) $$C_{k}\frac{dV_{k}}{dt}+g_{k}(t,\omega )V_{k}=i_{k}(t,\omega ),$$

where:

(17) $$g_{k}(t,\omega )=g_{L,k}+\sum_{j=1}^{N}G_{kj}\alpha_{kj}(t,\omega ).$$

This is the more general conductance form considered in this paper.

Moreover,

(18) $$i_{k}(t,\omega )=g_{L,k}E_{L}+\sum_{j=1}^{N}W_{kj}\alpha_{kj}(t,\omega )+i_{k}^{\left(\mathrm{ext}\right)}(t)+\sigma_{B}\xi_{k}(t),$$

where $W_{kj}$ is the synaptic weight:

$$\left\{ \begin{array}{ll} W_{kj}=E^{+}G_{kj}, & \text{if }j\in E,\\ W_{kj}=E^{-}G_{kj}, & \text{if }j\in I. \end{array}\right.$$

These equations hold when the membrane potential is below the threshold (Integrate regime).

Therefore, gIF models constitute rather complex dynamical systems: the vector field (r.h.s) of the differential Equation (16) depends on an auxiliary “variable”, which is the past spike sequence $\omega_{-\infty }^{\left[t\right]}$ and to define properly the evolution of $V_{k}$ from time *t* to later times one needs to know the spikes arising before *t*. This is precisely what makes gIF models more interesting than LIF. The definition of conductances introduces long-term *memory* effects.

IF models implement a reset mechanism on the membrane potential: If neuron *k* has been reset between *s* and *t*, say at time *τ*, then $V_{k}(t)$ depends only on $V_{k}(\tau )$ and not on previous values, as in (4). But, in gIF model, contrarily to LIF, there is also a dependence in the past via the conductance and this dependence *is not* erased by the reset. That is why we have to consider a system with infinite memory.

### 3.7 The parameters space

The stochastic dynamical system (16) depends on a huge set of parameters: the membrane capacities $C_{k}$, k=1,…,N, the threshold *θ*, the reversal potentials $E_{L}$, $E^{+}$, $E^{-}$, the leak conductance $g_{L}$; the maximal synaptic conductances $G_{kj}$, k,j=1,…,N which define the neural network topology; the characteristic times $\tau_{kj}$, k,j=1,…,N of synaptic responses decay; the noise amplitude $\sigma_{B}$; and additionally, the parameters defining the external current $i_{k}^{\left(\mathrm{ext}\right)}$. Although some parameters can be fixed from biology, such as $C_{k}$, the reversal potentials, $\tau_{kj}$, …some others such as the $G_{kj}$’s must be allowed to vary freely in order to leave open the possibility of modeling very different neural networks structures.

In this paper, we are not interested in describing properties arising for specific values of those parameters, but instead in generic properties that hold on sets of parameters. More specifically, we denote the list of all parameters $\left({\left(C_{k}\right)}_{k=1}^{N},E_{L},E^{+},E^{-},{\left(G_{kj}\right)}_{k,j=1}^{N},\ldots \right)$ by the symbol *γ*. This is a vector in $R^{K}$ where *K* is the total number of parameters. In this paper, we assume that *γ* belongs to a bounded subset $H\subset R^{K}$. Basically, we want to avoid situations where some parameters become infinite, which would be unphysical. So the limits of  are the limits imposed by biophysics. Additionally, we assume that $\sigma_{R}>0$ and $\sigma_{B}>0$. Together with physical constraints such as “conductances are positive”, these are the only assumption made in parameters. All mathematical results stated in the paper hold for any γ∈H.

## 4 gIF model dynamics for a fixed raster

We assume that the raster *ω* is fixed, namely the spike history is given. Then, it is possible to integrate the Equation (16) (Integrate regime) and to obtain explicitly the value of the membrane potential of a neuron at time *t*, given the membrane potential value at time *s*. Additionally, the reset condition (4) has the consequence of removing the dependence of neuron *k* on the past anterior to $\tau_{k}(t,\omega )$.

### 4.1 Integrate regime

For $t_{1}\leq t_{2}$, $t_{1},t_{2}\in R$, set:

(19) $$\Gamma_{k}(t_{1},t_{2},\omega )=e^{-\frac{1}{C_{k}}\int_{t_{1}}^{t_{2}}g_{k}(u,\omega )\;du}.$$

We have:

$$\Gamma_{k}(t_{1},t_{1},\omega )=\Gamma_{k}(t_{2},t_{2},\omega )=1,$$

and:

$$\frac{\partial \Gamma_{k}(t_{1},t_{2},\omega )}{\partial t_{1}}=\frac{g_{k}(t_{1},\omega )}{C_{k}}\Gamma_{k}(t_{1},t_{2},\omega ).$$

Fix two times s<t and assume that for neuron *k*, $V_{k}(u)<\theta$, s≤u≤t so that the membrane potential $V_{k}$ obeys (16). Then,

$$\begin{array}{rcl} \frac{\partial }{\partial t_{1}}\left[\Gamma_{k}(t_{1},t_{2},\omega )V_{k}(t_{1})\right] & = & \Gamma_{k}(t_{1},t_{2},\omega )\left[\frac{dV_{k}}{dt_{1}}+\frac{g_{k}(t_{1},\omega )}{C_{k}}V_{k}(t_{1})\right]\\ & = & \Gamma_{k}(t_{1},t_{2},\omega )\frac{i_{k}(t_{1},\omega )}{C_{k}}. \end{array}$$

We have then integrating the previous equation with respect to $t_{1}$ between *s* and *t* and setting $t_{2}=t$:

$$V_{k}(t)=\Gamma_{k}(s,t,\omega )V_{k}(s)+\frac{1}{C_{k}}\int_{s}^{t}\Gamma_{k}(t_{1},t,\omega )i_{k}(t_{1},\omega )\;dt_{1}.$$

This equation gives the variation in membrane potential during a period of rest (no spike) of the neuron. Note, however, that this neuron can still receive spikes from the other neurons via the update of conductances (made explicit in the previous equation by the dependence in the raster plot *ω*).

The term $\Gamma_{k}(s,t,\omega )$ given by (19) is an effective leak between s,t. In the leaky integrate and fire model, it would have been equal to $e^{-\int_{s}^{t}\frac{1}{\tau_{L}}dt_{1}}=e^{-\frac{t-s}{\tau_{L}}}$. The term:

$$V_{k}(s,t,\omega )\overset{\mathrm{def}}{=}\frac{1}{C_{k}}\int_{s}^{t}\Gamma_{k}(t_{1},t,\omega )i_{k}(t_{1},\omega )\;dt_{1},$$

has the dimension of a voltage. It corresponds to the integration of the total current between *s* and *t* weighted by the effective leak term $\Gamma_{k}(t_{1},t,\omega )$. It decomposes as

$$V_{k}(s,t,\omega )=V_{k}^{\left(\mathrm{syn}\right)}(s,t,\omega )+V_{k}^{\left(\mathrm{ext}\right)}(s,t,\omega )+V_{k}^{\left(B\right)}(s,t,\omega ),$$

where,

(20) $$V_{k}^{\left(\mathrm{syn}\right)}(s,t,\omega )=\frac{1}{C_{k}}\sum_{j=1}^{N}W_{kj}\int_{s}^{t}\Gamma_{k}(t_{1},t,\omega )\alpha_{kj}(t_{1},\omega )\;dt_{1},$$

is the synaptic contribution. Moreover,

$$V_{k}^{\left(\mathrm{ext}\right)}(s,t,\omega )=\frac{E_{L}}{\tau_{L,k}}\int_{s}^{t}\Gamma_{k}(t_{1},t,\omega )\;dt_{1}+\frac{1}{C_{k}}\int_{s}^{t}i_{k}^{\left(\mathrm{ext}\right)}(t_{1})\Gamma_{k}(t_{1},t,\omega )\;dt_{1},$$

where we set:

(21) $$\tau_{L,k}\overset{\mathrm{def}}{=}\frac{C_{k}}{g_{L,k}},$$

the characteristic leak time of neuron *k*. We have included the leak reversal potential term in this “external” term for convenience. Therefore, even if there is no external current, this term is nevertheless non-zero.

The sum of the synaptic and external terms gives the deterministic contribution in the membrane potential. We note:

$$V_{k}^{\left(\det\right)}(s,t,\omega )=V_{k}^{\left(\mathrm{syn}\right)}(s,t,\omega )+V_{k}^{\left(\mathrm{ext}\right)}(s,t,\omega ).$$

Finally,

(22) $$V_{k}^{\left(B\right)}(s,t,\omega )\overset{\mathrm{def}}{=}\frac{\sigma_{B}}{C_{k}}\int_{s}^{t}\Gamma_{k}(t_{1},t,\omega )\xi_{k}(t_{1})\;dt_{1}=\frac{\sigma_{B}}{C_{k}}\int_{s}^{t}\Gamma_{k}(t_{1},t,\omega )\;dB_{k}(t_{1}),$$

is a noise term. This is a Gaussian process with mean 0 and variance:

(23) $${\left(\frac{\sigma_{B}}{C_{k}}\right)}^{2}E\left[{\left(\int_{s}^{t}\Gamma_{k}(t_{1},t,\omega )\;dB_{k}(t_{1})\right)}^{2}\right]={\left(\frac{\sigma_{B}}{C_{k}}\right)}^{2}\int_{s}^{t}\Gamma_{k}^{2}(t_{1},t,\omega )\;dt_{1}.$$

The square root of this quantity has the dimension of a voltage.

As a final result, for a fixed *ω*, the variation in membrane potential during a period of rest (no spike) of neuron *k* between *s* and *t* reads (subthreshold oscillations):

(24) $$V_{k}(t)=\Gamma_{k}(s,t,\omega )V_{k}(s)+V_{k}^{\left(\det\right)}(s,t,\omega )+V_{k}^{\left(B\right)}(s,t,\omega ).$$

### 4.2 Reset

In Equation (4), as in all IF models that we know, the reset of the membrane potential has the effect of removing the dependence of $V_{k}$ on its past since $V_{k}([t+\tau_{\mathrm{sep}}])$ is replaced by $V_{\mathrm{reset}}$. Hence, reset removes the dependence in the initial condition $V_{k}(s)$ in (24) provided that neuron *k* fires between *s* and *t* in the raster *ω*. As a consequence, Equation (24) holds, from the “last reset time” introduced in Section 2.10 up to time *t*. Then, Equation (24) reads

(25) $$V_{k}(t)=V_{k}^{\left(\det\right)}\left(\tau_{k}(t,\omega ),t,\omega \right)+V_{k}^{\left(\mathrm{noise}\right)}\left(\tau_{k}(t,\omega ),t,\omega \right),$$

where:

(26) $$V_{k}^{\left(\mathrm{noise}\right)}\left(\tau_{k}(t,\omega ),t,\omega \right)=\Gamma_{k}\left(\tau_{k}(t,\omega ),t,\omega \right)V_{\mathrm{reset}}+V_{k}^{\left(B\right)}\left(\tau_{k}(t,\omega ),t,\omega \right),$$

is a Gaussian process with mean zero and variance:

(27) $$\sigma_{k}^{2}\left(\tau_{k}(t,\omega ),t,\omega \right)=\Gamma_{k}^{2}\left(\tau_{k}(t,\omega ),t,\omega \right)\sigma_{R}^{2}+{\left(\frac{\sigma_{B}}{C_{k}}\right)}^{2}\int_{\tau_{k}(t,\omega )}^{t}\Gamma_{k}^{2}(t_{1},t,\omega )\;dt_{1}.$$

## 5 Useful bounds

We now prove several bounds used throughout the paper.

### 5.1 Bounds on the conductance

From (13), and since $\alpha_{kj}(t)\geq 0$:

(28) $$\begin{array}{rcl} 0 & = & \alpha_{kj}(t,\Omega_{0})\leq \alpha_{kj}(t,\omega )=\sum_{\left\{r;t_{j}^{\left(r\right)}(\omega )<t\right\}}\alpha_{kj}\left(t-t_{j}^{\left(r\right)}(\omega )\right)\leq \sum_{r<t}\alpha_{kj}(t-r)\\ & = & \alpha_{kj}(t,\Omega_{1})\leq \alpha^{+}. \end{array}$$

Therefore,

(29) $$g_{L,k}=g_{k}(t,\Omega_{0})\leq g_{k}(t,\omega )\leq g_{k}(t,\Omega_{1})\overset{\mathrm{def}}{=}g_{M,k}\leq g_{L,k}+\alpha^{+}\sum_{j=1}^{N}G_{kj},$$

so that the conductance is uniformly bounded in *t* and *ω*. The minimal conductance is attained when no neuron fires ever so that Ω_0_ is the “lowest conductance state”. On the opposite, the maximal conductance is reached when all neurons fire all the time so that Ω_1_ is the “highest conductance state”. To simplify notations, we note $\tau_{M,k}=\frac{C_{k}}{g_{M,k}}$. This is the minimal relaxation time scale for neuron *k* while $\tau_{L,k}=\frac{C_{k}}{g_{L,k}}$ is the maximal relaxation time.

(30) $$\tau_{M,k}=\frac{C_{k}}{g_{M,k}}\leq \tau_{L,k}=\frac{C_{k}}{g_{L,k}}.$$

### 5.2 Bounds on membrane potential

Now, from (19), we have, for s<t:

(31) $$0\leq \Gamma_{k}(s,t,\Omega_{1})=e^{-\frac{t-s}{\tau_{M,k}}}\leq \Gamma_{k}(s,t,\omega )\leq \Gamma_{k}(s,t,\Omega_{0})=e^{-\frac{t-s}{\tau_{L,k}}}<1.$$

As a consequence, $\Gamma_{k}(s,t,\omega )\to 0$ exponentially fast as s→−∞.

Moreover,

(32) $$\begin{array}{rcl} 0 & \leq & \int_{s}^{t}\Gamma_{k}(t_{1},t,\omega )\alpha_{kj}(t_{1},\omega )\;dt_{1}\leq \alpha^{+}\int_{s}^{t}e^{-\frac{t-t_{1}}{\tau_{L,k}}}\;dt_{1}\\ & = & \alpha^{+}\tau_{L,k}\left(1-e^{-\frac{t-s}{\tau_{L,k}}}\right)\leq \alpha^{+}\tau_{L,k}, \end{array}$$

so that:

(33) $$\frac{\alpha^{+}}{g_{L,k}}\sum_{j\in I}W_{kj}\leq V_{k}^{\left(\mathrm{syn}\right)}(s,t,\omega )\leq \frac{\alpha^{+}}{g_{L,k}}\sum_{j\in E}W_{kj}.$$

Thus, $V_{k}^{\left(\mathrm{syn}\right)}(s,t,\omega )$ is uniformly bounded in s,t.

Establishing similar bounds for $V_{k}^{\left(\mathrm{ext}\right)}(s,t,\omega )$ requires the assumption that $A\leq i_{k}^{\left(\mathrm{ext}\right)}(t)\leq B$, but obtaining tighter bounds requires additionally the knowledge of the sign of $\frac{A}{C_{k}}+\frac{E_{L}}{\tau_{L,k}}$ and of $\frac{B}{C_{k}}+\frac{E_{L}}{\tau_{L,k}}$. Here, we have only to consider that:

$$\left|i_{k}^{\left(\mathrm{ext}\right)}(t)\right|\leq i^{+}.$$

In this case,

$$\begin{array}{rcl} \left|V_{k}^{\left(\mathrm{ext}\right)}(s,t,\omega )\right| & = & \left|\frac{E_{L}}{\tau_{L,k}}\int_{s}^{t}\Gamma_{k}(t_{1},t,\omega )\;dt_{1}+\frac{1}{C_{k}}\int_{s}^{t}i_{k}^{\left(\mathrm{ext}\right)}(t_{1})\Gamma_{k}(t_{1},t,\omega )\;dt_{1}\right|\\ & \leq & \left[\frac{\left|E_{L}\right|}{\tau_{L,k}}+\frac{i^{+}}{C_{k}}\right]\int_{s}^{t}\Gamma_{k}(t_{1},t,\omega )\;dt_{1}\leq \left[\frac{\left|E_{L}\right|}{\tau_{L,k}}+\frac{i^{+}}{C_{k}}\right]\int_{s}^{t}e^{-\frac{t-t_{1}}{\tau_{L,k}}}\;dt_{1}, \end{array}$$

so that:

(34) $$\left|V_{k}^{\left(\mathrm{ext}\right)}(s,t,\omega )\right|\leq \left(|E_{L}|+\frac{i^{+}}{g_{L,k}}\right)\left(1-e^{-\frac{t-s}{\tau_{L,k}}}\right)\leq |E_{L}|+\frac{i^{+}}{g_{L,k}}.$$

Consequently,

**Proposition 2**

(35) $$\begin{array}{rcl} V_{k}^{-} & \overset{\mathrm{def}}{=} & \frac{\alpha^{+}}{g_{L,k}}\sum_{j\in I}W_{kj}-\left(|E_{L}|+\frac{i^{+}}{g_{L,k}}\right)<V_{k}^{\left(\det\right)}(s,t,\omega )<V_{k}^{+}\\ & \overset{\mathrm{def}}{=} & \frac{\alpha^{+}}{g_{L,k}}\sum_{j\in E}W_{kj}+|E_{L}|+\frac{i^{+}}{g_{L,k}}, \end{array}$$

*which provides uniform bounds ins*, *t*, *ωfor the deterministic part of the membrane potential*.

### 5.3 Bounds on the noise variance

Let us now consider the stochastic part $V_{k}^{\left(\mathrm{noise}\right)}(\tau_{k}(t,\omega ),t,\omega )$. It has zero mean, and its variance (27) obeys the bounds:

$$\begin{array}{rcl} & & e^{-2\frac{t-\tau_{k}(t,\omega )}{\tau_{M,k}}}\sigma_{R}^{2}+\frac{\tau_{M,k}}{2}{\left(\frac{\sigma_{B}}{C_{k}}\right)}^{2}\left(1-e^{-2\frac{t-\tau_{k}(t,\omega )}{\tau_{M,k}}}\right)\\ & & \;\leq \sigma_{k}^{2}\left(\tau_{k}(t,\omega ),t,\omega \right)\\ & & \;\leq e^{-2\frac{t-\tau_{k}(t,\omega )}{\tau_{L,k}}}\sigma_{R}^{2}+\frac{\tau_{L,k}}{2}{\left(\frac{\sigma_{B}}{C_{k}}\right)}^{2}\left(1-e^{-2\frac{t-\tau_{k}(t,\omega )}{\tau_{L,k}}}\right). \end{array}$$

If $\sigma_{R}^{2}<\frac{\tau_{M,k}}{2}{\left(\frac{\sigma_{B}}{C_{k}}\right)}^{2}$ the left-hand side is an increasing function of $u=t-\tau_{k}(t,\omega )\geq 0$ so that the minimum, $\sigma_{R}^{2}$ is reached for u=0 while the maximum is reached for u=+∞ and is $\frac{\tau_{M,k}}{2}{\left(\frac{\sigma_{B}}{C_{k}}\right)}^{2}$. The opposite holds if $\sigma_{R}^{2}\geq \frac{\tau_{M,k}}{2}{\left(\frac{\sigma_{B}}{C_{k}}\right)}^{2}$. The same argument holds *mutatis mutandis* for the right-hand side. We set:

(36) $$\sigma_{k}^{-}\overset{\mathrm{def}}{=}\min\left(\frac{\sigma_{B}}{C_{k}}\sqrt{\frac{\tau_{M,k}}{2}},\sigma_{R}\right);\;\sigma_{k}^{+}\overset{\mathrm{def}}{=}\max\left(\frac{\sigma_{B}}{C_{k}}\sqrt{\frac{\tau_{L,k}}{2}},\sigma_{R}\right)$$

so that:

**Proposition 3**

(37) $$0<\sigma_{k}^{-}\leq \sigma_{k}\left(\tau_{k}(t,\omega ),t,\omega \right)\leq \sigma_{k}^{+}<+\infty .$$

### 5.4 The limit $\tau_{k}(t,\omega )\to -\infty$

For fixed *s* and *t*, there are infinitely many rasters such that $\tau_{k}(t,\omega )<s$ (we remind that rasters are infinite sequences). One may argue that taking the difference t−s sufficiently large, the probability of such sequences should vanish. It is indeed possible to show (Section 8.1) that this probability vanishes exponentially fast with t−s, meaning unfortunately that it is *positive* whatever t−s. So we have to consider cases where $\tau_{k}(t,\omega )$ can go arbitrary far in past (this is also a key toward an extension of the present analysis to more general conductance-based models as discussed in Section 9.3). Therefore, we have to check that the quantities introduced in the previous sections are well defined as $\tau_{k}(t,\omega )\to -\infty$.

Fix *s* real. For all *ω* such that $\tau_{k}(t,\omega )\leq s$ - this condition ensuring that *k* does not fire between *s* and *t* - we have, from (28), (31), $0\leq \Gamma_{k}(t_{1},t,\omega )\alpha_{kj}(t_{1},\omega )\leq \alpha^{+}e^{-\frac{t-t_{1}}{\tau_{L,k}}}$, $\forall t_{1}\in [s,t]$. Now, since $\lim_{s\to -\infty }\int_{s}^{t}e^{-\frac{t-t_{1}}{\tau_{L,k}}}\;dt_{1}=\tau_{L,k}$ exists, the limit

$$\lim_{\tau_{k}(t,\omega )\to -\infty }V_{k}^{\left(\mathrm{syn}\right)}\left(\tau_{k}(t,\omega ),t,\omega \right)=\frac{1}{C_{k}}\sum_{j=1}^{N}W_{kj}\int_{-\infty }^{t}\Gamma_{k}(t_{1},t,\omega )\alpha_{kj}(t_{1},\omega )\;dt_{1}.$$

exists as well. The same holds for the external term $V_{k}^{\left(\mathrm{ext}\right)}(\tau_{k}(t,\omega ),t,\omega )$.

Finally, since $\Gamma_{k}(\tau_{k}(t,\omega ),t,\omega )\to 0$ as $\tau_{k}(t,\omega )\to -\infty$ the noise term (26) becomes in the limit:

$$\lim_{\tau_{k}(t,\omega )\to -\infty }V_{k}^{\left(\mathrm{noise}\right)}\left(\tau_{k}(t,\omega ),t,\omega \right)=\frac{\sigma_{B}}{C_{k}}\int_{-\infty }^{t}\Gamma_{k}(t_{1},t,\omega )\;dB_{k}(t_{1}),$$

which is a Gaussian process with mean 0 and a variance ${\left(\frac{\sigma_{B}}{C_{k}}\right)}^{2}\int_{-\infty }^{t}\Gamma_{k}^{2}(t_{1},t,\omega )\;dt_{1}$ which obeys the bounds (37).

## 6 Continuity with respect to a raster

### 6.1 Definition

Due to the particular structure of gIF models, we have seen that the membrane potential at time *t* is both a function of *t* and of the full sequence of past spikes $\omega_{-\infty }^{\left[t\right]}$. One expects, however, the dependence with respect to the past spikes to decay as those spikes are more distant in the past. This issue is related to a notion of continuity with respect to a raster that we now characterize.

**Definition 1** Let *m* be a positive integer. The *m*-*variation* of a function $f(t,\omega )\equiv f(t,\omega_{-\infty }^{\left[t\right]})$ is:

(38) $${\mathrm{var}}_{m}[f(t,\cdot )]=\sup\left\{\left|f(t,\omega )-f\left(t,\omega^{\prime }\right)\right|:\omega \overset{m,[t]}{=}\omega^{\prime }\right\}.$$

where the definition of $\overset{m,[t]}{=}$ is given in Equation (5). Hence, this notion characterizes the maximal variation of f(t,⋅) on the set of spikes identical from time [t]−m to time [t] (cylinder set). It implements the fact that one may truncate the spike history to time [t]−m and make an error which is at most ${\mathrm{var}}_{m}[f(t,\cdot )]$.

**Definition 2** The function f(t,ω) is *continuous* if ${\mathrm{var}}_{m}[f(t,\cdot )]\to 0$ as m→+∞.

An additional information is provided by the convergence rate to 0 with *m*. The faster this convergence, the smaller the error made when replacing an infinite raster by a spike block on a finite time horizon.

### 6.2 Continuity of conductances

**Proposition 4***The conductance*$g_{k}(t,\omega )$*is continuous inω*, *for allt*, *for all*k=1,…,N.

*Proof* Fix k=1,…,N, t∈R, m>0 integer. We have, for $\omega \overset{m,[t]}{=}\omega^{\prime }$:

$$\begin{array}{rcl} & & \left|\alpha_{kj}(t,\omega )-\alpha_{kj}\left(t,\omega^{\prime }\right)\right|\\ & & \;=\left|\sum_{\left\{r;t_{j}^{\left(r\right)}(\omega )<t\right\}}\alpha_{kj}\left(t-t_{j}^{\left(r\right)}(\omega )\right)-\sum_{\left\{r^{\prime };t_{j}^{\left(r^{\prime }\right)}(\omega^{\prime })<t\right\}}\alpha_{kj}\left(t-t_{j}^{\left(r^{\prime }\right)}\left(\omega^{\prime }\right)\right)\right|\\ & & \;=\left|\sum_{\left\{r;t_{j}^{\left(r\right)}(\omega )<t-m\right\}}\alpha_{kj}\left(t-t_{j}^{\left(r\right)}(\omega )\right)-\sum_{\left\{r^{\prime };t_{j}^{\left(r^{\prime }\right)}(\omega^{\prime })<t-m\right\}}\alpha_{kj}\left(t-t_{j}^{\left(r^{\prime }\right)}\left(\omega^{\prime }\right)\right)\right|, \end{array}$$

since the set of firing times $\left\{t-m\leq t_{j}^{\left(r\right)}(\omega )<t\right\}$, $\left\{t-m\leq t_{j}^{\left(n^{\prime }\right)}(\omega^{\prime })<t\right\}$ are identical by hypothesis. So, since $\alpha_{kj}(x)\geq 0$,

$$\begin{array}{rcl} & & \left|\alpha_{kj}(t,\omega )-\alpha_{kj}\left(t,\omega^{\prime }\right)\right|\\ & & \;\leq \sum_{\left\{r;t_{j}^{\left(r\right)}(\omega )<t-m\right\}}\alpha_{kj}\left(t-t_{j}^{\left(r\right)}(\omega )\right)+\sum_{\left\{r^{\prime };t_{j}^{\left(r^{\prime }\right)}(\omega^{\prime })<t-m\right\}}\alpha_{kj}\left(t-t_{j}^{\left(r^{\prime }\right)}\left(\omega^{\prime }\right)\right)\\ & & \;\leq 2\sum_{r<t-m}\alpha_{kj}(t-r). \end{array}$$

Therefore, as m→+∞, from (12) and setting M=t−m,

$${\mathrm{var}}_{m}\left[\alpha_{kj}(t,\cdot )\right]\leq 2\sum_{r<M}\alpha_{kj}(t-r)⪯2P_{d}\left(\frac{m}{\tau_{kj}}\right)e^{-\frac{m}{\tau_{kj}}},$$

which converges to 0 as m→+∞.

Therefore, from (17), $g_{k}(t,\omega )$ is continuous with a variation

$${\mathrm{var}}_{m}\left[g_{k}(t,\cdot )\right]⪯2\sum_{j=1}^{N}G_{kj}P_{d}\left(\frac{m}{\tau_{kj}}\right)e^{-\frac{m}{\tau_{kj}}},$$

which converges exponentially fast to 0 as m→+∞. □

### 6.3 Continuity of the membrane potentials

**Proposition 5***The deterministic part of the membrane potential*, $V_{k}^{\left(\det\right)}(\tau_{k}(t,\omega ),t,\omega )$, *is continuous and itsm*-*variation decays exponentially fast withm*.

*Proof* In the proof, we shall establish precise upper bounds for the variation in $V_{k}^{\left(\mathrm{syn}\right)}(\tau_{k}(t,\cdot ),t,\cdot )$, $V_{k}^{\left(\mathrm{ext}\right)}(\tau_{k}(t,\cdot ),t,\cdot )$ since they are used later on for the proof of uniqueness of a Gibbs measure (Section 7.4.1). From the previous result, it is easy to show that, for all $\omega \overset{m,[t]}{=}\omega^{\prime }$, $t_{1}\leq t_{2}\leq t$:

$$\left|\Gamma_{k}(t_{1},t_{2},\omega )-\Gamma_{k}\left(t_{1},t_{2},\omega^{\prime }\right)\right|\leq \Gamma_{k}(t_{1},t_{2},\omega )\left(e^{\frac{{\mathrm{var}}_{m}[g_{k}]}{C_{k}}(t_{2}-t_{1})}-1\right),$$

Therefore, from (31),

$${\mathrm{var}}_{m}\left[\Gamma_{k}(t_{1},t_{2},\cdot )\right]\leq e^{-\frac{t_{2}-t_{1}}{\tau_{L,k}}}\left(e^{\frac{{\mathrm{var}}_{m}[g_{k}]}{C_{k}}(t_{2}-t_{1})}-1\right)$$

and $\Gamma_{k}(t_{1},t_{2},\omega )$ is continuous in *ω*.

Now, the product $\Gamma_{k}(t_{1},t_{2},\omega )\alpha_{kj}(t_{1},\omega )$ is continuous as a product of continuous functions. Moreover,

$$\begin{array}{rcl} & & {\mathrm{var}}_{m}\left[\Gamma_{k}(t_{1},t_{2},\cdot )\alpha_{kj}(t_{1},\cdot )\right]\\ & & \;\leq \underset{\omega \in X}{\sup}\Gamma_{k}(t_{1},t_{2},\omega ){\mathrm{var}}_{m}\left[\alpha_{kj}(t_{1},\cdot )\right]+\underset{\omega \in X}{\sup}\alpha_{kj}(t_{1},\omega ){\mathrm{var}}_{m}\left[\Gamma_{k}(t_{1},t_{2},\cdot )\right]\\ & & \;=e^{-\frac{t_{2}-t_{1}}{\tau_{L,k}}}\left[{\mathrm{var}}_{m}\left[\alpha_{kj}(t_{1},\cdot )\right]+\left(e^{\frac{{\mathrm{var}}_{m}[g_{k}]}{C_{k}}(t_{2}-t_{1})}-1\right)\alpha_{kj}(t_{1},\Omega_{1})\right], \end{array}$$

so that:

$${\mathrm{var}}_{m}\left[\Gamma_{k}(t_{1},t_{2},\cdot )\alpha_{kj}(t_{1},\cdot )\right]<e^{-\frac{t_{2}-t_{1}}{\tau_{L,k}}}\left[2P_{d}\left(\frac{m}{\tau_{kj}}\right)e^{-\frac{m}{\tau_{kj}}}+\left(e^{\frac{{\mathrm{var}}_{m}[g_{k}]}{C_{k}}(t_{2}-t_{1})}-1\right)\alpha^{+}\right].$$

Since, as m→+∞:

$$\begin{array}{rcl} e^{\frac{{\mathrm{var}}_{m}[g_{k}]}{C_{k}}(t_{2}-t_{1})}-1 & ∼ & \frac{{\mathrm{var}}_{m}[g_{k}]}{C_{k}}(t_{2}-t_{1})\\ & ⪯ & \frac{2(t_{2}-t_{1})}{C_{k}}\sum_{j^{\prime }=1}^{N}G_{kj^{\prime }}P_{d}\left(\frac{m}{\tau_{kj^{\prime }}}\right)e^{-\frac{m}{\tau_{kj^{\prime }}}}, \end{array}$$

we have,

$$\begin{array}{rl} & {\mathrm{var}}_{m}\left[\Gamma_{k}(t_{1},t_{2},\cdot )\alpha_{kj}(t_{1},\cdot )\right]\\ & \;⪯2e^{-\frac{t_{2}-t_{1}}{\tau_{L,k}}}\left[P_{d}\left(\frac{m}{\tau_{kj}}\right)e^{-\frac{m}{\tau_{kj}}}+\frac{\alpha^{+}(t_{2}-t_{1})}{C_{k}}\sum_{j^{\prime }=1}^{N}G_{kj^{\prime }}P_{d}\left(\frac{m}{\tau_{kj^{\prime }}}\right)e^{-\frac{m}{\tau_{kj^{\prime }}}}\right], \end{array}$$

which converges to 0 as m→+∞.

Let us show the continuity of $V_{k}^{\left(\mathrm{syn}\right)}(\tau_{k}(t,\cdot ),t,\cdot )$. We have, from (20),

$$\begin{array}{rcl} & & \left|V_{k}^{\left(\mathrm{syn}\right)}\left(\tau_{k}(t,\omega ),t,\omega \right)-V_{k}^{\left(\mathrm{syn}\right)}\left(\tau_{k}\left(t,\omega^{\prime }\right),t,\omega^{\prime }\right)\right|\\ & & \;\leq \frac{1}{C_{k}}\sum_{j=1}^{N}|W_{kj}|\\ & & \;\times \left|\int_{\tau_{k}(t,\omega )}^{t}\Gamma_{k}(t_{1},t,\omega )\alpha_{kj}(t_{1},\omega )\;dt_{1}-\int_{\tau_{k}(t,\omega^{\prime })}^{t}\Gamma_{k}\left(t_{1},t,\omega^{\prime }\right)\alpha_{kj}\left(t_{1},\omega^{\prime }\right)\;dt_{1}\right|. \end{array}$$

The following inequality is used at several places in the paper. For a $t_{1}$-integrable function $f(t_{1},t,\omega )$, we have:

(39) $$\begin{array}{rcl} & & \left|\int_{\tau_{k}(t,\omega )}^{t}f(t_{1},t,\omega )\;dt_{1}-\int_{\tau_{k}(t,\omega^{\prime })}^{t}f(t_{1},t,\omega^{\prime })\;dt_{1}\right|\\ & & \;\leq \int_{\tau_{k}(t,\omega )}^{t}\left|f(t_{1},t,\omega )-f\left(t_{1},t,\omega^{\prime }\right)\right|\;dt_{1}+\left|\int_{\tau_{k}(t,\omega )}^{\tau_{k}(t,\omega^{\prime })}f\left(t_{1},t,\omega^{\prime }\right)\;dt_{1}\right|. \end{array}$$

Here, it gives, for $t_{1}\leq t$:

$$\begin{array}{rcl} & & \left|\int_{\tau_{k}(t,\omega )}^{t}\Gamma_{k}(t_{1},t,\omega )\alpha_{kj}(t_{1},\omega )\;dt_{1}-\int_{\tau_{k}(t,\omega^{\prime })}^{t}\Gamma_{k}\left(t_{1},t,\omega^{\prime }\right)\alpha_{kj}\left(t_{1},\omega^{\prime }\right)\;dt_{1}\right|\\ & & \;\leq \int_{\tau_{k}(t,\omega )}^{t}\left|\Gamma_{k}(t_{1},t,\omega )\alpha_{kj}(t_{1},\omega )-\Gamma_{k}\left(t_{1},t,\omega^{\prime }\right)\alpha_{kj}\left(t_{1},\omega^{\prime }\right)\right|\;dt_{1}\\ & & \;+\left|\int_{\tau_{k}(t,\omega )}^{\tau_{k}(t,\omega^{\prime })}\Gamma_{k}\left(t_{1},t,\omega^{\prime }\right)\alpha_{kj}\left(t_{1},\omega^{\prime }\right)\;dt_{1}\right|. \end{array}$$

For the first term, we have,

$$\begin{array}{rcl} & & \int_{\tau_{k}(t,\omega )}^{t}\left|\Gamma_{k}(t_{1},t,\omega )\alpha_{kj}(t_{1},\omega )-\Gamma_{k}\left(t_{1},t,\omega^{\prime }\right)\alpha_{kj}\left(t_{1},\omega^{\prime }\right)\right|\;dt_{1}\\ & & \;\leq \int_{\tau_{k}(t,\omega )}^{t}{\mathrm{var}}_{m}\left[\Gamma_{k}(t_{1},t,\cdot )\alpha_{kj}(t_{1},\cdot )\right]\;dt_{1}\leq \int_{-\infty }^{t}{\mathrm{var}}_{m}\left[\Gamma_{k}(t_{1},t,\cdot )\alpha_{kj}(t_{1},\cdot )\right]\;dt_{1}\\ & & \;⪯\int_{-\infty }^{t}2e^{-\frac{t-t_{1}}{\tau_{L,k}}}\left[P_{d}\left(\frac{m}{\tau_{kj}}\right)e^{-\frac{m}{\tau_{kj}}}+\frac{\alpha^{+}(t-t_{1})}{C_{k}}\sum_{j^{\prime }=1}^{N}G_{kj^{\prime }}P_{d}\left(\frac{m}{\tau_{kj^{\prime }}}\right)e^{-\frac{m}{\tau_{kj^{\prime }}}}\right]\;dt_{1}\\ & & \;=2P_{d}\left(\frac{m}{\tau_{kj}}\right)e^{-\frac{m}{\tau_{kj}}}\int_{-\infty }^{t}e^{-\frac{t-t_{1}}{\tau_{L,k}}}\;dt_{1}\\ & & \;+\frac{2\alpha^{+}}{C_{k}}\sum_{j^{\prime }=1}^{N}G_{kj^{\prime }}P_{d}\left(\frac{m}{\tau_{kj^{\prime }}}\right)e^{-\frac{m}{\tau_{kj^{\prime }}}}\int_{-\infty }^{t}(t-t_{1})e^{-\frac{t-t_{1}}{\tau_{L,k}}}\;dt_{1}\\ & & \;=2\tau_{L,k}\left[P_{d}\left(\frac{m}{\tau_{kj}}\right)e^{-\frac{m}{\tau_{kj}}}+\frac{\alpha^{+}\tau_{L,k}}{C_{k}}\sum_{j^{\prime }=1}^{N}G_{kj^{\prime }}P_{d}\left(\frac{m}{\tau_{kj^{\prime }}}\right)e^{-\frac{m}{\tau_{kj^{\prime }}}}\right]. \end{array}$$

Let us now consider the second term. If $\tau_{k}(t,\omega )\geq t-m$ or $\tau_{k}(t,\omega^{\prime })\geq t-m$, then $\tau_{k}(t,\omega )=\tau_{k}(t,\omega^{\prime })$ and this term vanishes. Therefore, the supremum in the definition of ${\mathrm{var}}_{m}[V_{k}^{\left(\mathrm{syn}\right)}(\tau_{k}(t,\cdot ),t,\cdot )]$ is attained if $\tau_{k}(t,\omega )<t-m$ and $\tau_{k}(t,\omega^{\prime })<t-m$. We may assume, without loss of generality, that $\tau_{k}(t,\omega^{\prime })\geq \tau_{k}(t,\omega )$. Then, from (32),

$$\begin{array}{rcl} & & \int_{\tau_{k}(t,\omega )}^{\tau_{k}(t,\omega^{\prime })}\Gamma_{k}\left(t_{1},t,\omega^{\prime }\right)\alpha_{kj}\left(t_{1},\omega^{\prime }\right)\;dt_{1}\\ & & \;<\alpha^{+}\int_{\tau_{k}(t,\omega )}^{\tau_{k}(t,\omega^{\prime })}e^{-\frac{t-t_{1}}{\tau_{L,k}}}\;dt_{1}=\alpha^{+}\tau_{L,k}e^{-\frac{t-\tau_{k}(t,\omega^{\prime })}{\tau_{L,k}}}\left(1-e^{-\frac{\tau_{k}(t,\omega^{\prime })-\tau_{k}(t,\omega )}{\tau_{L,k}}}\right)\\ & & \;\leq \alpha^{+}\tau_{L,k}e^{-\frac{t-\tau_{k}(t,\omega^{\prime })}{\tau_{L,k}}}\leq \alpha^{+}\tau_{L,k}e^{-\frac{m}{\tau_{L,k}}}. \end{array}$$

So, we have, for the variation of $V_{k}^{\left(\mathrm{syn}\right)}(\tau_{k}(t,\cdot ),t,\cdot )$, using (21):

varm[Vk(syn)(τk(t,⋅),t,⋅)]⪯1gL,k∑j=1N|Wkj|[2(Pd(mτkj)e−mτkj+α+gL,k∑j′=1NGkj′Pd(mτkj′)e−mτkj′)1gL,k∑j=1N|Wkj|[+α+e−mτL,k],

so that finally,

(40) $${\mathrm{var}}_{m}\left[V_{k}^{\left(\mathrm{syn}\right)}\left(\tau_{k}(t,\cdot ),t,\cdot \right)\right]⪯\sum_{j=1}^{N}A_{kj}^{\left(\mathrm{syn}\right)}P_{d}\left(\frac{m}{\tau_{kj}}\right)e^{-\frac{m}{\tau_{kj}}}+B_{k}^{\left(\mathrm{syn}\right)}e^{-\frac{m}{\tau_{L,k}}},$$

with

(41) $$A_{kj}^{\left(\mathrm{syn}\right)}=\frac{1}{g_{L,k}}\left(2|W_{kj}|+\alpha^{+}\frac{G_{kj}}{g_{L,k}}\sum_{j^{\prime }=1}^{N}|W_{kj^{\prime }}|\right),$$

(42) $$B_{k}^{\left(\mathrm{syn}\right)}=\frac{\alpha^{+}}{g_{L,k}}\sum_{j=1}^{N}|W_{kj}|,$$

and ${\mathrm{var}}_{m}[V_{k}^{\left(\mathrm{syn}\right)}(\tau_{k}(t,\cdot ),t,\cdot )]$ converges to 0 exponentially fast as m→+∞.

Now, let us show the continuity of $V_{k}^{\left(\mathrm{ext}\right)}(\tau_{k}(t,\omega ),t,\omega )$ with respect to *ω*. We have:

|Vk(ext)(τk(t,ω),t,ω)−Vk(ext)(τk(t,ω′),t,ω′)|=|∫τk(t,ω)t(ELτL,k+ik(ext)(t1)Ck)Γk(t1,t,ω)dt1|−∫τk(t,ω′)t(ELτL,k+ik(ext)(t1)Ck)Γk(t1,t,ω′)dt1|≤∫τk(t,ω)t|ELτL,k+ik(ext)(t1)Ck||Γk(t1,t,ω)−Γk(t1,t,ω′)|dt1+∫τk(t,ω)τk(t,ω′)|ELτL,k+ik(ext)(t1)Ck|Γk(t1,t,ω′)dt1≤(|EL|τL,k+i+Ck)×(∫τk(t,ω)t|Γk(t1,t,ω)−Γk(t1,t,ω′)|dt1+∫τk(t,ω)τk(t,ω′)Γk(t1,t,ω′)dt1)≤(|EL|τL,k+i+Ck)(∫τk(t,ω)tvarm[Γk(t1,t,⋅)]dt1+∫τk(t,ω)τk(t,ω′)e−t−t1τL,kdt1)⪯(|EL|τL,k+i+Ck)(2τL,k2Ck∑j′=1NGkj′Pd(mτkj′)e−mτkj′+τL,ke−mτL,k),

where, in the last inequality, we have used that the supremum in the variation is attained for $\tau_{k}(t,\omega )<t-m$ and $\tau_{k}(t,\omega^{\prime })<t-m$. Finally:

(43) $${\mathrm{var}}_{m}\left[V_{k}^{\left(\mathrm{ext}\right)}\left(\tau_{k}(t,\cdot ),t,\cdot \right)\right]⪯\sum_{j=1}^{N}A_{kj}^{\left(\mathrm{ext}\right)}P_{d}\left(\frac{m}{\tau_{kj}}\right)e^{-\frac{m}{\tau_{kj}}}+B_{k}^{\left(\mathrm{ext}\right)}e^{-\frac{m}{\tau_{L,k}}},$$

where,

(44) $$A_{kj}^{\left(\mathrm{ext}\right)}=2\frac{G_{kj}}{g_{L,k}}B_{k}^{\left(\mathrm{ext}\right)},$$

(45) $$B_{k}^{\left(\mathrm{ext}\right)}=|E_{L}|+\frac{i^{+}}{g_{L,k}},$$

and $V_{k}^{\left(\mathrm{ext}\right)}(\tau_{k}(t,\cdot ),t,\cdot )$ is continuous.

As a conclusion, $V_{k}^{\left(\det\right)}(\tau_{k}(t,\cdot ),t,\cdot )$ is continuous as the sum of two continuous functions. □

### 6.4 Continuity of the variance of $V_{k}^{\left(\mathrm{noise}\right)}(\tau_{k}(t,\cdot ),t,\cdot )$

Using the same type of arguments, one can also prove that

**Proposition 6***The variance*$\sigma_{k}(\tau_{k}(t,\omega ),t,\omega )$*is continuous inω*, *for allt*, *for all*k=1,…,N.

*Proof* We have, from (27)

$$\begin{array}{rcl} & & \left|\sigma_{k}^{2}\left(\tau_{k}(t,\omega ),t,\omega \right)-\sigma_{k}^{2}\left(\tau_{k}\left(t,\omega^{\prime }\right),t,\omega^{\prime }\right)\right|\\ & & \;\leq \sigma_{R}^{2}{\mathrm{var}}_{m}\left[\Gamma_{k}^{2}\left(\tau_{k}(t,\cdot ),t,\cdot \right)\right]\\ & & \;+{\left(\frac{\sigma_{B}}{C_{k}}\right)}^{2}\left|\int_{\tau_{k}(t,\omega )}^{t}\Gamma_{k}^{2}(t_{1},t,\omega )\;dt_{1}-\int_{\tau_{k}(t,\omega^{\prime })}^{t}\Gamma_{k}^{2}\left(t_{1},t,\omega^{\prime }\right)\;dt_{1}\right|. \end{array}$$

For the first term, we have that the sup in ${\mathrm{var}}_{m}[\Gamma_{k}^{2}(\tau_{k}(t,\cdot ),t,\cdot )]$ is attained for $\tau_{k}(t,\omega ),\tau_{k}(t,\omega^{\prime })<t-m$ and:

$${\mathrm{var}}_{m}\left[\Gamma_{k}^{2}\left(\tau_{k}(t,\cdot ),t,\cdot \right)\right]\leq 2\sup\left\{\Gamma_{k}^{2}\left(\tau_{k}(t,\omega ),t,\omega \right);\omega \;\text{s.t.}\;\tau_{k}(t,\omega )<t-m\right\}\leq 2e^{-\frac{2m}{\tau_{L,k}}}.$$

For the second term, we have:

$$\begin{array}{rcl} & & \left|\int_{\tau_{k}(t,\omega )}^{t}\Gamma_{k}^{2}(t_{1},t,\omega )\;dt_{1}-\int_{\tau_{k}(t,\omega^{\prime })}^{t}\Gamma_{k}^{2}\left(t_{1},t,\omega^{\prime }\right)\;dt_{1}\right|\\ & & \;\leq \int_{\tau_{k}(t,\omega )}^{t}\left|\Gamma_{k}^{2}(t_{1},t,\omega )-\Gamma_{k}^{2}\left(t_{1},t,\omega^{\prime }\right)\right|\;dt_{1}+\int_{\tau_{k}(t,\omega )}^{\tau_{k}(t,\omega^{\prime })}\Gamma_{k}^{2}\left(t_{1},t,\omega^{\prime }\right)\;dt_{1}\\ & & \;\leq \int_{\tau_{k}(t,\omega )}^{t}{\mathrm{var}}_{m}\left[\Gamma_{k}^{2}(t_{1},t,\cdot )\right]\;dt_{1}+\int_{\tau_{k}(t,\omega )}^{\tau_{k}(t,\omega^{\prime })}e^{-\frac{2(t-t_{1})}{\tau_{L,k}}}\;dt_{1}\\ & & \;\leq \int_{-\infty }^{t}e^{-\frac{2(t-t_{1})}{\tau_{L,k}}}\left(e^{\frac{2{\mathrm{var}}_{m}[g_{k}(t,\cdot )(t-t_{1})]}{C_{k}}}-1\right)\;dt_{1}+\frac{\tau_{L,k}}{2}e^{-\frac{2m}{\tau_{L,k}}}\\ & & \;⪯\frac{\tau_{L,k}^{2}}{2C_{k}}{\mathrm{var}}_{m}\left[g_{k}(t,\cdot )\right]+\frac{\tau_{L,k}}{2}e^{-\frac{2m}{\tau_{L,k}}}, \end{array}$$

so that finally,

(46) $${\mathrm{var}}_{m}\left[\sigma_{k}^{2}(t,\cdot )\right]⪯\sum_{j=1}^{N}A_{kj}^{\left(\sigma \right)}P_{d}\left(\frac{m}{\tau_{kj}}\right)e^{-\frac{m}{\tau_{kj}}}+C_{k}^{\left(\sigma \right)}e^{-\frac{2m}{\tau_{L,k}}},$$

with

$$\begin{array}{rcl} A_{kj}^{\left(\sigma \right)} & = & \frac{G_{kj}}{g_{L,k}}{\left(\frac{\sigma_{B}\sqrt{\tau_{L,k}}}{C_{k}}\right)}^{2},\\ C_{k}^{\left(\sigma \right)} & = & \frac{1}{2}{\left(\frac{\sigma_{B}\sqrt{\tau_{L,k}}}{C_{k}}\right)}^{2}+2\sigma_{R}^{2}, \end{array}$$

and continuity follows. □

### 6.5 Remark

Note that the variation in all quantities considered here is exponentially decaying with a time constant given by $\max(\tau_{kj},\tau_{L,k})$. This is physically satisfactory: the loss of memory in the system is controlled by the leak time and the decay of the post-synaptic potential.

## 7 Statistics of raster plots

### 7.1 Conditional probability distribution of $V_{k}(t)$

Recall that *P* is the joint distribution of the noise and E[] the expectation under *P*. Under *P*, the membrane potential *V* is a stochastic process whose evolution, below the threshold, is given Equations (24), (25) and above by (4). It follows from the previous analysis that:

**Proposition 7***Conditionally to*$\omega_{-\infty }^{\left[t\right]}$, V(t)*is Gaussian with mean*:

$$E\left[V_{k}(t)|\omega_{-\infty }^{\left[t\right]}\right]=V_{k}^{\left(\det\right)}\left(\tau_{k}(t,\omega ),t,\omega \right),\;k=1,\ldots ,N,$$

*and covariance*:

$$\mathrm{Cov}\left[V_{k}(t),V_{l}(t)|\omega_{-\infty }^{\left[t\right]}\right]=\sigma_{k}^{2}\left(\tau_{k}(t,\omega ),t,\omega \right)\delta_{kl},\;k,l=1,\ldots ,N$$

*where*$\sigma_{k}^{2}(\tau_{k}(t,\omega ),t,\omega )$*is given by* (27).

*Moreover*, *the*$V_{k}(t)$*’s*, k=1,…,N*are conditionally independent*.

*Proof* Essentially, the proof is a direct consequence of Equations (24), (25) and the Gaussian nature of the noise $V_{k}^{\left(\mathrm{noise}\right)}(\tau_{k}(t,\omega ),t,\omega )$. The conditional independence results from the fact that:

Cov[Vk(t),Vl(t)|ω−∞[t]]=σB2CkClE[∫τk(t,ω)tΓk(t1,t,ω)dBk(t1)∫τl(t,ω)tΓl(t2,t,ω)dBl(t2)|ω]+Cov[Γk(τk(t,ω),t,ω)Vreset,Γl(τl(t,ω),t,ω)Vreset]=σB2CkCl∫τk(t,ω)t∫τl(t,ω)tΓk(t1,t,ω)Γl(t2,t,ω)E[dBk(t1)dBl(t2)]+σR2Γk2(τk(t,ω),t,ω)δkl=δkl[(σBCk)2∫τk(t,ω)t∫τk(t,ω)tΓk(t1,t,ω)Γk(t2,t,ω)δ(t1−t2)dt1dt2δkl[+σR2Γk2(τk(t,ω),t,ω)]=σk2(τk(t,ω),t,ω)δkl.

 □

### 7.2 The transition probability

We now compute the probability of a spiking pattern at time n=[t], ω(n), given the past sequence $\omega_{-\infty }^{n-1}$.

**Proposition 8***The probability of*ω(n)*conditionally to*$\omega_{-\infty }^{n-1}$*is given by*:

(47) $$P\left[\omega (n)|\omega_{-\infty }^{n-1}\right]=\prod_{k=1}^{N}P\left[\omega_{k}(n)|\omega_{-\infty }^{n-1}\right],$$

*with*

(48) $$\begin{array}{rcl} P\left[\omega_{k}(n)|\omega_{-\infty }^{n-1}\right] & = & \omega_{k}(n)\pi \left(X_{k}(n-1,\omega )\right)\\ & & +\left(1-\omega_{k}(n)\right)\left(1-\pi \left(X_{k}(n-1,\omega )\right)\right), \end{array}$$

*where*

(49) $$X_{k}(n-1,\omega )=\frac{\theta -V_{k}^{\left(\det\right)}(\tau_{k}(n-1,\omega ),n-1,\omega )}{\sigma_{k}(\tau_{k}(n-1,\omega ),n-1,\omega )},$$

*and*

(50) $$\pi (x)=\frac{1}{\sqrt{2\pi }}\int_{x}^{+\infty }e^{-\frac{u^{2}}{2}}\;du.$$

*Proof* We have, using the conditional independence of the $V_{k}(n)$’s:

$$\begin{array}{rl} & P\left[\omega (n)|\omega_{-\infty }^{n-1}\right]\\ & \;=\prod_{k=1}^{N}\left(\omega_{k}(n)P\left[V_{k}(n-1)\geq \theta |\omega_{-\infty }^{n-1}\right]+\left(1-\omega_{k}(n)\right)P\left[V_{k}(n-1)<\theta |\omega_{-\infty }^{n-1}\right]\right). \end{array}$$

Since the $V_{k}(n-1)$’s are conditionally Gaussian, with mean $V_{k}^{\left(\det\right)}(\tau_{k}(n-1,\omega ),n-1,\omega )$ and variance $\sigma_{k}^{2}(\tau_{k}(n-1,\omega ),n-1,\omega )$, we directly obtain (47), (48).

Note that since $\sigma_{k}(\tau_{k}(n-1,\omega ),n-1,\omega )$ is bounded from below by a positive quantity (see (37)), the ratio $\frac{\theta -V_{k}^{\left(\det\right)}(\tau_{k}(n-1,\omega ),n-1,\omega )}{\sigma_{k}(\tau_{k}(n-1,\omega ),n-1,\omega )}$ in (48) is defined for all ω∈X. □

### 7.3 Chains with complete connections

 The transition probabilities (47) define a stochastic process on the set of raster plots where the underlying membrane potential dynamics is summarized in the terms $V_{k}^{\left(\det\right)}(\tau_{k}(n-1,\omega ),n-1,\omega )$ and $\sigma_{k}(\tau_{k}(n-1,\omega ),n-1,\omega )$. While the integral defining these terms extends from $\tau_{k}(n-1,\omega )$ to n−1 where $\tau_{k}(n-1,\omega )$ can go arbitrary far in the past, the integrand involves the conductance $g_{k}(n-1,\omega )$ that summarizes an history dating back to s=−∞. As a consequence, the probability transitions generate a stochastic process with unbounded memory, thus non-Markovian. One may argue that this property is a result of our procedure of taking the initial condition in a infinite past s→−∞, to remove the unresolved dependency on $g_{k}(s)$ (Section 3.4). So the alternative is either to keep *s* finite in order to have a Markovian process; then, we have to fix arbitrarily $g_{k}(s)$ and the probability distribution of $V_{k}(s)$. Or we take s→−∞, removing the initial condition, to the price of considering a non-Markovian process. Actually, such processes are widely studied in the literature under the name of “chains with complete connections” [43,46-49] and several important results can be used here. So we adopt the second approach of the alternative. As a by-product, the knowledge of the Gibbs measure provided by this analysis allows a posteriori to fix the probability distribution of $V_{k}(s)$.

 For the sake of completeness, we give here the definition of a chain with complete connections (see [43] for more details). For n∈Z, we note $A_{-\infty }^{n-1}$ the set of sequences $\omega_{-\infty }^{n-1}$ and $F_{\leq n-1}$ the related *σ*-algebra, while  is the *σ*-algebra related with $X=A^{Z}$. P(X,F) is the set of probability measures on (X,F).

**Definition 3** A system of transition probabilities is a family ${\left\{P_{n}\right\}}_{n\in Z}$ of functions

$$P_{n}[|]:A\times A_{-\infty }^{n-1}\to [0,1],$$

such that the following conditions hold for every n∈Z: 

• For every ω(n)∈A, the function $P_{n}[\omega (n)|.]$ is measurable with respect to $F_{\leq n-1}$.

• For every $\omega_{-\infty }^{n-1}\in A_{-\infty }^{n-1}$,

$$\sum_{\omega (n)\in A}P_{n}\left[\omega (n)|\omega_{-\infty }^{n-1}\right]=1.$$

A probability measure *μ* in P(X,F) is consistent with a system of transition probabilities ${\left\{P_{n}\right\}}_{n\in Z}$ if for all n∈Z and all $F_{\leq n}$-measurable functions *f*:

$$\int f\left(\omega_{-\infty }^{n}\right)\mu (d\omega )=\int \sum_{\omega (n)\in A}f\left(\omega_{-\infty }^{n-1}\omega (n)\right)P_{n}\left[\omega (n)|\omega_{-\infty }^{n-1}\right]\mu (d\omega ).$$

Such a measure *μ* is called a “chain with complete connections consistent with the system of transition probabilities ${\left\{P_{n}\right\}}_{n\in Z}$”.

The transitions probabilities (47) constitute such a system of transitions probabilities: the summation to 1 is obvious while the measurability follows from the continuity of $P_{n}[\omega (n)|\omega_{-\infty }^{n-1}]$ proved below. To simplify notations, we write p(n,ω) instead of $P_{n}[\omega (n)|\omega_{-\infty }^{n-1}]$ whenever it makes no confusion.

### 7.4 Existence of a consistent probability measure *μ*

 In the definition above, the measure *μ* summarizes the statistics of spike trains from −∞ to +∞. Its marginals allow the characterization of finite spike blocks. So, *μ* provides the characterization of spike train statistics in gIF models. Its existence is established by a standard result in the frame of chains with complete connections stating that a system of continuous transition probabilities on a compact space has at least one probability measure consistent with it [43]. Since *π* is a continuous function the continuity of p(n,ω) with respect to *ω* follows from the continuity of $V_{k}^{\left(\det\right)}(\tau_{k}(n-1,\omega ),n-1,\omega )$ and the continuity of $\sigma_{k}(\tau_{k}(n-1,\omega ),n-1,\omega )$, proved in Section 6.

Therefore, there is at least one probability measure consistent with (47).

#### 7.4.1 The Gibbs distribution

 A system of transition probabilities is non-null if for all n∈Z and all $\omega_{-\infty }^{n-1}\in A_{-\infty }^{n-1}$$P[\omega (n)|\omega_{-\infty }^{n-1}]>0$. Following [50], a chain with complete connection *μ* is a Gibbs measure consistent with the system of transition probabilities p(n,⋅) if this system is continuous and non-null. Gibbs distributions play an important role in statistical physics, as well as ergodic theory and stochastic processes. In statistical physics, they are usually derived from the maximal entropy principle [14]. Here, we use them in a more general context affording to consider non-stationary processes. It turns out that the spike train statistics in gIF model is given by such a Gibbs measure. In this section, we prove the main mathematical result of this paper (uniqueness of the Gibbs measure). The consequences for spike trains characterizations are discussed in the next section.

**Theorem 1***For each choice of parameters*γ∈H, *the gIF model* (16) *has a unique Gibbs distribution*.

 The proof of uniqueness is based on the following criteria due to Fernandez and Maillard [50].

**Proposition 9***Let*:^2^

$$m(p)=\underset{n\in Z}{\inf}\underset{\omega \in A_{-\infty }^{n}}{\inf}p(n,\omega ),$$

and

$$v(p)=\underset{m^{\prime }\in Z}{\sup}\sum_{n\geq m^{\prime }}{\mathrm{var}}_{n-m^{\prime }}\left[p(n,\cdot )\right].$$

*If*m(p)>0*and*v(p)<∞, *then there exists at most one Gibbs measure consistent with it*.

So, to prove the uniqueness, we only have to establish that

(51) m(p)>0,

(52) v(p)<+∞.

*Proof*m(p)>0.

Recall that:

$$p(n,\omega )=\prod_{k=1}^{N}\left[\omega_{k}(n)\pi \left(X_{k}(n-1,\omega )\right)+\left(1-\omega_{k}(n)\right)\left(1-\pi \left(X_{k}(n-1,\omega )\right)\right)\right].$$

From (35), (37), we have:

(53) $$-\infty <\frac{\theta -V_{k}^{+}}{\sigma_{k}^{+}}<X_{k}(n-1,\omega )<\frac{\theta -V_{k}^{-}}{\sigma_{k}^{-}}<+\infty .$$

Since *π*, given by (50), is monotonously decreasing, we have:

$$0<\pi_{k}^{-}\overset{\mathrm{def}}{=}\pi \left(\frac{\theta -V_{k}^{-}}{\sigma_{k}^{-}}\right)<\pi \left(X_{k}(n-1,\omega )\right)<\pi_{k}^{+}\overset{\mathrm{def}}{=}\pi \left(\frac{\theta -V_{k}^{+}}{\sigma_{k}^{+}}\right)<1,$$

so that:

(54) $$\begin{array}{rcl} 0 & < & \Pi_{k}^{-}\overset{\mathrm{def}}{=}\min\left(\pi_{k}^{-},1-\pi_{k}^{+}\right)<\omega_{k}(n)\pi_{k}^{-}+\left(1-\omega_{k}(n)\right)\left(1-\pi_{k}^{+}\right)<p_{k}(n,\omega )\\ & < & \omega_{k}(n)\pi_{k}^{+}+\left(1-\omega_{k}(n)\right)\left(1-\pi_{k}^{-}\right)<\Pi_{k}^{+}\overset{\mathrm{def}}{=}\max\left(\pi_{k}^{+},1-\pi_{k}^{-}\right)<1. \end{array}$$

Finally,

$$m(p)>\prod_{k=1}^{N}\Pi_{k}^{-}>0,$$

which proves (51). This also proves the non-nullness of the system of transition probabilities.

v(p)<∞.

The proof, which is rather long, is given in the appendix. □

## 8 Consequences

### 8.1 The probability that neuron *k* does not fire in the time interval [s,t]

In Section 4.2, we argued that this probability vanishes exponentially fast with t−s. This probability is $\mu [{⋂}_{n=[s]+1}^{\left[t\right]}\{\omega_{k}(n)=0\}]$. We now prove this result.

**Proposition 10***The probability that neuronkdoes not fire within the time interval*[s,t], t−s>1*has the following bounds*:

$$0<\Pi_{-}^{\left[t]-[s\right]}<\mu \left[\overset{\left[t\right]}{\underset{n=[s]+1}{⋂}}\left\{\omega_{k}(n)=0\right\}\right]<\Pi_{+}^{\left[t]-[s\right]}<1,$$

*for some constants*$0<\Pi_{-}<\Pi_{+}<1$*depending on the system parameters*γ∈H.

*Proof* We have:

$$\begin{array}{rcl} \mu \left[\overset{\left[t\right]}{\underset{n=[s]+1}{⋂}}\left\{\omega_{k}(n)=0\right\}\right] & = & \int_{A_{-\infty }^{\left[s\right]}}\mu \left[\overset{\left[t\right]}{\underset{n=[s]+1}{⋂}}\left\{\omega_{k}(n)=0\right\}|\omega_{-\infty }^{\left[s\right]}\right]\;d\mu (\omega )\\ & = & \int_{A_{-\infty }^{\left[s\right]}}\prod_{n=[s]+1}^{\left[t\right]}\mu \left[\left\{\omega_{k}(n)=0\right\}|\omega_{-\infty }^{n-1}\right]\;d\mu (\omega )\\ & = & \int_{A_{-\infty }^{\left[s\right]}}\prod_{n=[s]+1}^{\left[t\right]}P\left[\omega_{k}(n)=0|\omega_{-\infty }^{n-1}\right]\;d\mu (\omega ), \end{array}$$

where $P[\omega_{k}(n)=0|\omega_{-\infty }^{n-1}]$ is given by (47) and obeys the bounds (54). Therefore, setting $\Pi_{-}=\prod_{k=1}^{N}\Pi_{k}^{-}$ and $\Pi^{+}=\prod_{k=1}^{N}\Pi_{k}^{+}$, we have

$$\begin{array}{rl} \Pi_{-}^{\left[t]-[s\right]}\int_{A_{-\infty }^{\left[s\right]}}\;d\mu (\omega ) & =\Pi_{-}^{\left[t]-[s\right]}\leq \mu \left[\tau_{k}(t,\omega )\leq s\right]\leq \Pi_{+}^{\left[t]-[s\right]}\int_{A_{-\infty }^{\left[s\right]}}\;d\mu (\omega )\\ & =\Pi_{+}^{\left[t]-[s\right]}. \end{array}$$

 □

### 8.2 Back to spike trains analysis with the maximal entropy principle

 Here, we shortly develop the consequences of our results in relation with the statistical model estimation discussed in the introduction. A more detailed discussion will be published elsewhere (in preparation and [51]). Set:

(55) $$\phi (n,\omega )\overset{\mathrm{def}}{=}\log p(n,\omega )=\sum_{k=1}^{N}\phi_{k}(n,\omega ),$$

with,

(56) $$\begin{array}{rcl} \phi_{k}(n,\omega ) & \overset{\mathrm{def}}{=} & \omega_{k}(n)\log\pi \left(X_{k}(n-1,\omega )\right)\\ & & \;+\left(1-\omega_{k}(n)\right)\log\left(1-\pi \left(X_{k}(n-1,\omega )\right)\right). \end{array}$$

The function *ϕ* is a Gibbs potential [52]. Indeed, we have ∀m<n$\forall \omega_{-\infty }^{n}$:

$$\mu \left[\omega_{m}^{n}|\omega_{-\infty }^{m-1}\right]=\exp\sum_{l=m}^{n}\phi (l,\omega ).$$

This equation emphasizes the connection with Gibbs distributions in statistical physics that considers probability distributions on multidimensional lattices with specified boundary conditions and their behavior under space translations [52]. The correspondence with our case is that “time” is represented by a mono-dimensional space and where the “boundary conditions” are the past $\omega_{-\infty }^{m-1}$. Note that in our case, the partition function is equal to 1.

For simplicity, assume stationarity (this is equivalent to assuming a time-independent external current). In this case, it is sufficient to consider the potential at time n=0.

Thanks to the bounds (53), one can make a series expansion of the functions log(π) and log(1−π) and rewrite the potential under the form of the expansion: (57)

 where P(N,R) is the set of non-repeated pairs of integers (k,N) with k∈{1,…,N} and n∈{−R,…,0}. We call the product $\omega_{k_{1}}(n_{1})\cdots \omega_{k_{r}}(n_{r})$ a *monomial*. It is 1 if and only if neuron $k_{1}$ fires at time $n_{1},\ldots ,k_{r}$ fires at time $n_{r}$. The $\lambda_{\left(k_{1},n_{1}),\ldots ,(k_{r},n_{r}\right)}$’s are explicit functions of the parameters *γ*. Due to the causal form of the potential, where the time-0 spike, $\omega_{k}(0)$, is multiplied by a function of the past $\omega_{-\infty }^{-1}$, the polynomial expansion does not contain monomials of the form $\omega_{k_{1}}(0)\cdots \omega_{k_{r}}(0)$, r>1 (the corresponding coefficient *λ* vanishes).

Since the potential has infinite range, the expansion (57) contains infinitely many terms. One can nevertheless consider truncations to a range R=D+1 corresponding to truncating the memory of the process to some memory depth *D*. Note that although truncations with a memory depth *D* are approximations, the distance with the exact potential converges exponentially fast to 0 as D→+∞ thanks to the continuity of the potential, with a decay rate controlled by synaptic responses and leak rate.

The truncated Gibbs potential has the form:

(58) $$\phi^{\left(D\right)}\left(\omega_{-D}^{0}\right)=\sum_{l}\lambda_{l}\phi_{l}\left(\omega_{-D}^{0}\right);$$

where *l* stands for $\left(k_{1},n_{1}),\ldots ,(k_{r},n_{r}\right)$ and is an enumeration of the elements in P(N,D+1) and where $\phi_{l}$ is the corresponding monomial. Due to the truncation, (58), contrarily to (55), is not normalized. Its partition function^3^ is not equal to 1, and its computation becomes rapidly intractable as soon as the number of neurons and memory depth increases.

Clearly, (58) *is precisely the form of potential which is obtained under the maximal entropy principle, where the*$\phi_{l}$*’s are constraints of type “neuron*$k_{1}$*is firing at time*$n_{1}$*, neuron*$k_{2}$*is firing at time*$n_{2},\ldots$*” and the*$\lambda_{l}$*’s the conjugated Lagrange multipliers*. Thus, using the maximal entropy principle to characterize spike statistics in the gIF model by expressing constraints in terms of spike events (monomials), one can at best find an approximation which can be rather bad, especially if those constraints focus on instantaneous spike patterns (D=0) or short memory patterns. Moreover, increasing the memory depth to approach better the right statistics leads to an exponential increase in the number of monomials which becomes rapidly intractable. Finally, the Lagrange multipliers $\lambda_{l}$ are rather difficult to interpret.

On the opposite, the analytic form (55) depends only on a finite numbers of parameters (*γ*) constraining the neural network dynamics, which have a straightforward interpretations being physical quantities. This shows that, at least in gIF model, the linear Gibbs potential (58) obtained from the maximal entropy principle is not really appropriate, even for empirical/numerical purposes, and that a form (55) where the infinite memory $\omega_{-\infty }^{-1}$ is replaced by $\omega_{-D}^{-1}$ could be more efficient although non-linear.

To finish this section, let us discuss the link with Ising model in light of the present work. Ising model corresponds to a memory-less case, hence to D=0. Since the causal structure of the Gibbs potential forbids monomials of the form $\omega_{k_{1}}(0)\cdots \omega_{k_{r}}(0)$, the D=0 expansion of the gIF-Gibbs potential corresponds to a *Bernoulli distribution where neurons are independent*$\phi^{\left(0\right)}(0,\omega )=\sum_{k=1}^{N}\lambda_{k}\omega_{k}(0)$. The Ising model is therefore irrelevant to approximate the exact potential of gIF model, if one wants to reproduce spike statistics at the *minimal discretization time scaleδ* without considering memory effects.

However, in real data analysis, people are usually *binning* data, with a time windows of width w∼10-20 ms. Binning consists of recoding the raster plot with spikes amalgamation. The binned raster *b* consists of “spikes” $b_{k}(n)\in \{0,1\}$ where $b_{k}(n)=1$ if neuron *k* fired *at least once* in the time window [nw,(n+1)w[. In the expansion (57), this corresponds to collecting all monomials corresponding to $b_{k}(n)=1$ in a unique monomial. In this way, the binned potential contains *indeed* an Ising term… that mixes all spike events occurring within the time interval *w*. These events appear simultaneous because of binning, leading to the Ising pairwise term $b_{k_{1}}(0)b_{k_{2}}(0)$ while events occurring on smaller time scales are scrambled by this procedure.

The binning effect on Gibbs potential requires, however, a more detailed description. This will be discussed elsewhere.

## 9 Discussion

To conclude this paper, we would like to discuss several consequences and possible extensions of this work.

### 9.1 The spike time discretization

In gIF model, membrane potential evolves continuously while conductance is updated with spike occurrence considered as discrete events. Here, we discuss this time discretization. Actually, there are two distinct questions.

#### 9.1.1 The limit of time-bin tending to 0

 This limit would correspond to a case where spike is instantaneous and modeled by a Dirac distribution. As discussed in [32], this limit raises serious difficulties. To summarize, in real neurons, firing occurs within a finite time *δ* corresponding to the time of raise and fall for the membrane potential. This involves physicochemical processes that cannot be instantaneous. The time curse of the membrane potential during the spike is described by differential equations, like Hodgkin-Huxley’s [35]. Although, the time scale d*t* appearing in the differential equations has the mathematical meaning of being arbitrary small, on biophysical grounds, this time scale cannot be arbitrary small, otherwise the Hodgkin-Huxley equations loose their meaning. Indeed, they correspond to an average over microscopic phenomena such as ionic channels dynamics. In particular, their time scale must be sufficiently *large* to ensure that the description of ionic channels dynamics (opening and closing) in terms of *probabilities* is valid so d*t* must be larger than the characteristic time of opening-closing of ionic channels $\tau_{P}$. Additionally, Hodgkin-Huxley’s equations use a Markovian approach (master equation) for the dynamics of *hmn* gates. This requires that the characteristic time d*t* is quite a bit larger than the characteristic time of decay for the time correlations between gates activity $\tau_{C}$. Summarizing, we must have $0<\tau_{C}$$\tau_{P}<dt<\delta$. Thus, on biophysical grounds, *δ* cannot be arbitrary small.

 In our case, the δ→0 limit is armless, however, provided we keep a non-zero refractory period, ensuring that only finitely many spikes occur in a finite time interval. Taking the limit δ→0 without considering a refractory period raises mathematical problems. One can in principle have uncountably many spikes in a finite time interval leading to the divergence of physical quantities like energy. Also, one can generate nice causal paradoxes [37]. Take a loop with two neurons one excitatory and one inhibitory and assume instantaneous propagation (the *α* profile is then represented by a Dirac distribution). Then, depending on the synaptic weights value, one can have a situation where neuron 1 fires instantaneously and make instantaneously 2 firing which prevents instantaneously 1 from firing and so on. So taking the limit δ→0 as well as $\tau_{\mathrm{refr}}\to 0$ induces pathologies not inherent to our approach but to IF models.

#### 9.1.2 Synchronization for distinct neurons

 There is a more subtle issue pointed out in [53]. We do not only discretize time for each neuron’ spikes, we align the spikes emitted by distinct neurons on a discrete-time grid, as an experimental raster does. As shown in [32] this induces, in gIF models with a purely deterministic dynamics (no noise and reset to a *constant value*), an artificial synchronization. As a consequence, the deterministic dynamics of gIF models has generically only stable periodic orbits, although periods can be larger than any accessible computational time in a specific region of the parameters space. Additionally, these periods increase as *δ* decreases. The addition of noise on dynamics and on the reset value, as we propose in this paper, removes this synchronization effect.

### 9.2 Refractory period

In the definition of the model, we have assumed that the refractory period $\tau_{\mathrm{ref}}$ was smaller than 1. The consequence for raster plots is that one can have two consecutive 1’s in the spike sequence of a neuron. The extension to the case where $\tau_{\mathrm{ref}}>1$ is straightforward for spike statistics. Having such a refractory period forbids some sequences. For example, if $1<\tau_{\mathrm{ref}}\leq 2$, then all sequences containing two consecutive 1’s for one neuron (1,1) are forbidden. If $2<\tau_{\mathrm{ref}}\leq 3$ sequences containing 1,∗,1 for a given neuron, where ∗=0,1, are forbidden, and so on. More generally, the procedure consisting of forbidding specific (finite) spike blocks is equivalent to introducing a *grammar* in the spike generation. This grammar can be implemented in the Gibbs potential: forbidden sequences have a potential equal to −∞ (resp. a zero probability). In this case, $X=A^{Z}$, the set of all possible rasters, becomes a subset where forbidden sequences have been removed.

### 9.3 Beyond IF models

 Let us now discuss the extension of the present work to more general models of neurons. First, one characteristic feature of integrate and fire models is the reset, which has the consequence that the memory of activity preceding the spike is lost after reset. Although in the deterministic (noiseless) case, this is a simplifying feature allowing for example to fully characterize the asymptotic dynamics of (discrete time) IF models [32,54], here, it somewhat renders more complex the analysis. Indeed, it led us to introduce the notion of “last reset” time and, at some point in the proof (see, e.g., Equation (39)), obliged us to consider several situations (e.g., $\tau_{k}(t,\omega )\geq t-m$ or $\tau_{k}(t,\omega^{\prime })\geq t-m$ versus $\tau_{k}(t,\omega )<t-m$ and $\tau_{k}(t,\omega^{\prime })<t-m$ in the proof of continuity, Section 6). On the opposite, considering a model where no such reset occur would simply lead us to consider a model where $\tau_{k}(t,\omega )\to -\infty$, ∀*ω*, ∀*k*. This case is already considered in our formalism, and actually, considering that $\tau_{k}(t,\omega )\to -\infty$, ∀*ω*, ∀*k* simplifies the proofs (for example it eliminates the second term in Equation (39)).

 As a matter of fact, the theorems established in this paper should therefore also hold without reset. But this requires to replace the firing condition (4) by another condition stating what the membrane potential does during the spike. Although it could be possible to propose an ad hoc form for the spike, it would certainly be more interesting to extend the results here to models where neurons activity depends on additional variables such as adaptation currents, as in the FitzHugh-Nagumo model [39-42], or activation-inactivation variables as in the Hodgkin-Huxley model [35].

 The present formalism affords an extension toward such models, where the neuron fires whenever its membrane potential belongs to a region of the phase space, which can be delimited by membrane potentials plus additional variables such as adaptation currents or activation-inactivation variables, and where the spike is controlled by the global dynamics of all these variables. But while here the firing of a neuron is described by the crossing of a fixed threshold, in the FitzHugh-Nagumo model, it is given by the crossing of a separatrix in the plane (voltage-adaptation current) and by a more complex “frontier” in the Hodgkin-Huxley model [2,55]. One difficulty is to precisely define this region. To our knowledge, there is no clear agreement for the Hodgkin-Huxley model (some authors [2] even suggest that the “spike region” could have a fractal frontier). The extension toward FitzHugh-Nagumo seems more manageable.

 Finally, the most important difficulty toward extending this paper results in more realistic neural networks is the definition of the synaptic spike response. In IF models, the spike is thought as a punctual “event” (typically, an “instantaneous” pulse) while the synaptic response is described by a convolution kernel (the *α*-profile). This leads one to consider a somewhat artificial mixed dynamics where membrane potential evolves continuously while spike are discrete events. In more realistic models, one would have to consider kinetic equations for neurotransmitter release, receptor binding and opening of post-synaptic ionic channels [5,44]. Additionally, the consideration of these mechanics deserves a spatially extended modeling of the neuron, with time delays. In this case, all variables evolve continuously and the statistics of spike trains would be characterized by the statistics of return times in the “spike region”. This statistics is induced by some probability measure in the phase space; a natural candidate would be the Sinai-Ruelle-Bowen measure [56-58], for stationary dynamics, or the time-dependent SRB measure for non-stationary cases, as defined, e.g., in [59]. These measures are Gibbs measures as well [60]. Here, the main mathematical property ensuring existence and uniqueness of such a measure would be uniform hyperbolicity. To our knowledge, conditions ensuring such a property in networks have not been established yet neither for Hodgkin-Huxley’s nor for FitzHugh-Nagumo’s models.

### 9.4 Synaptic plasticity

 As the results established in this paper hold for any synaptic weight value in , they hold as well for networks underlying synaptic plasticity mechanisms. The effects of a joint evolution of spikes dynamics, depending on synaptic weights distributions, and synaptic weights evolution depending on spike dynamics have been studied in [61]. In particular it has been shown that mechanisms such as Spike-Time-Dependent Plasticity are related to a variational principle for a quantity, the topological pressure, derived for the thermodynamic formalism of Gibbs distributions. In the paper [61], the fact that spike trains statistics were given by a Gibbs distribution was a working assumption. Therefore, the present work establishes a firm ground for [61].

## Appendix

Here, we establish (52). We use the following lemma.

**Lemma 1***For a collection*$0\leq a_{k},a_{k}^{\prime }\leq 1$, ∀k=1,…,N, *we have*

(59) $$\left|\prod_{k=1}^{N}a_{k}-\prod_{k=1}^{N}a_{k}^{\prime }\right|\leq \sum_{k=1}^{N}\left|a_{k}-a_{k}^{\prime }\right|.$$

This lemma is easily proved by recursion.

We have, for n∈Z, m≥0

$${\mathrm{var}}_{m}\left[p(n,\cdot )\right]=\sup\left\{\left|\prod_{k=1}^{N}a_{k}-\prod_{k=1}^{N}a_{k}^{\prime }\right|;\omega \overset{m,n}{=}\omega^{\prime }\right\},$$

where:

$$\begin{array}{rcl} a_{k} & = & \omega_{k}(n)\pi \left(X_{k}(n-1,\omega )\right)+\left(1-\omega_{k}(n)\right)\left(1-\pi \left(X_{k}(n-1,\omega )\right)\right),\\ a_{k}^{\prime } & = & \omega_{k}^{\prime }(n)\pi \left(X_{k}\left(n-1,\omega^{\prime }\right)\right)+\left(1-\omega_{k}^{\prime }(n)\right)\left(1-\pi \left(X_{k}\left(n-1,\omega^{\prime }\right)\right)\right). \end{array}$$

Therefore, using inequality (59),

$${\mathrm{var}}_{m}\left[p(n,\cdot )\right]\leq \sum_{k=1}^{N}\sup\left\{\left|a_{k}-a_{k}^{\prime }\right|;\omega \overset{m,n}{=}\omega^{\prime }\right\}.$$

The condition $\omega \overset{m,n}{=}\omega^{\prime }$ implies $\omega_{k}(n)=\omega_{k}^{\prime }(n)$ so that:

$$\left|a_{k}-a_{k}^{\prime }\right|=\left|\pi \left(X_{k}(n-1,\omega )\right)-\pi \left(X_{k}\left(n-1,\omega^{\prime }\right)\right)\right|.$$

We have

$$\left|\pi \left(X_{k}(n-1,\omega )\right)-\pi \left(X_{k}\left(n-1,\omega^{\prime }\right)\right)\right|\leq \left|X_{k}(n-1,\omega )-X_{k}\left(n-1,\omega^{\prime }\right)\right|{∥\pi^{\prime }∥}_{\infty },$$

with ${∥\pi^{\prime }∥}_{\infty }=\sup_{x\in R}|\pi^{\prime }(x)|=\frac{1}{\sqrt{2\pi }}$, so that

$${\mathrm{var}}_{m}\left[p(n,\cdot )\right]\leq \frac{1}{\sqrt{2\pi }}\sum_{k=1}^{N}{\mathrm{var}}_{m}\left[X_{k}(n-1,\cdot )\right].$$

We have now to upper bound ${\mathrm{var}}_{m}[X_{k}(n-1,\cdot )]=\sup\{|X_{k}(n-1,\omega )-X_{k}(n-1,\omega^{\prime })|;\omega \overset{m,n-1}{=}\omega^{\prime }\}$. We have

$$\begin{array}{rcl} & & {\mathrm{var}}_{m}\left[X_{k}(n-1,\cdot )\right]\\ & & \;\leq {\mathrm{var}}_{m}\left[\theta -V_{k}^{\left(\det\right)}\left(\tau_{k}(n-1,\cdot ),n-1,\cdot \right)\right]\underset{\omega \in X}{\sup}\frac{1}{\sigma_{k}(\tau_{k}(n-1,\omega ),n-1,\omega )}\\ & & \;+\underset{\omega \in X}{\sup}\left|\theta -V_{k}^{\left(\det\right)}\left(\tau_{k}(n-1,\omega ),n-1,\omega \right)\right|{\mathrm{var}}_{m}\left[\frac{1}{\sigma_{k}(\tau_{k}(n-1,\cdot ),n-1,\cdot )}\right], \end{array}$$

with,

$$\begin{array}{rcl} & & {\mathrm{var}}_{m}\left[\theta -V_{k}^{\left(\det\right)}\left(\tau_{k}(n-1,\cdot ),n-1,\cdot \right)\right]={\mathrm{var}}_{m}\left[V_{k}^{\left(\det\right)}\left(\tau_{k}(n-1,\cdot ),n-1,\cdot \right)\right]\\ & & \;\leq {\mathrm{var}}_{m}\left[V_{k}^{\left(\mathrm{syn}\right)}\left(\tau_{k}(n-1,\cdot ),n-1,\cdot \right)\right]+{\mathrm{var}}_{m}\left[V_{k}^{\left(\mathrm{ext}\right)}\left(\tau_{k}(n-1,\cdot ),n-1,\cdot \right)\right] \end{array}$$

so that, from (40), (43):

(60) $$\begin{array}{rl} & {\mathrm{var}}_{m}\left[\theta -V_{k}^{\left(\det\right)}\left(\tau_{k}(n-1,\cdot ),n-1,\cdot \right)\right]\\ & \;⪯\sum_{j=1}^{N}A_{kj}^{\left(\det\right)}P_{d}\left(\frac{m}{\tau_{kj}}\right)e^{-\frac{m}{\tau_{kj}}}+B_{k}^{\left(\det\right)}e^{-\frac{m}{\tau_{L,k}}} \end{array}$$

where:

$$A_{kj}^{\left(\det\right)}=A_{kj}^{\left(\mathrm{syn}\right)}+A_{kj}^{\left(\mathrm{ext}\right)},$$

(see Equations (41), (44)) and:

$$B_{k}^{\left(\det\right)}=B_{k}^{\left(\mathrm{syn}\right)}+B_{k}^{\left(\mathrm{ext}\right)},$$

(see Equations (42), (45)).

Moreover, from (37),

(61) $$\underset{\omega \in X}{\sup}\frac{1}{\sigma_{k}(\tau_{k}(n-1,\omega ),n-1,\omega )}\leq \frac{1}{\sigma_{k}^{-}}.$$

From (35),

(62) $$\underset{\omega \in X}{\sup}\left|\theta -V_{k}^{\left(\det\right)}\left(\tau_{k}(n-1,\omega ),n-1,\omega \right)\right|\leq \max\left(\left|\theta -V_{k}^{-}\right|,\left|\theta -V_{k}^{+}\right|\right).$$

Finally,

$$\begin{array}{rcl} & & {\mathrm{var}}_{m}\left[\frac{1}{\sigma_{k}(\tau_{k}(n-1,\cdot ),n-1,\cdot )}\right]\\ & & \;\leq {\mathrm{var}}_{m}\left[\sigma_{k}\left(\tau_{k}(n-1,\cdot ),n-1,\cdot \right)\right]\underset{\omega \in X}{\sup}\frac{1}{\sigma_{k}^{2}(\tau_{k}(n-1,\omega ),n-1,\omega )}\\ & & \;\leq \frac{1}{{\left(\sigma_{k}^{-}\right)}^{2}}{\mathrm{var}}_{m}\left[\sigma_{k}\left(\tau_{k}(n-1,\cdot ),n-1,\cdot \right)\right], \end{array}$$

while

$${\mathrm{var}}_{m}\left[\sigma_{k}^{2}\left(\tau_{k}(n-1,\cdot ),n-1,\cdot \right)\right]\geq 2\sigma_{k}^{-}{\mathrm{var}}_{m}\left[\sigma_{k}\left(\tau_{k}(n-1,\cdot ),n-1,\cdot \right)\right],$$

from (37), so that:

$${\mathrm{var}}_{m}\left[\sigma_{k}\left(\tau_{k}(n-1,\cdot ),n-1,\cdot \right)\right]\leq \frac{1}{2\sigma_{k}^{-}}{\mathrm{var}}_{m}\left[\sigma_{k}^{2}\left(\tau_{k}(n-1,\cdot ),n-1,\cdot \right)\right],$$

and, from (46),

(63) $$\begin{array}{rcl} & & {\mathrm{var}}_{m}\left[\frac{1}{\sigma_{k}(\tau_{k}(n-1,\cdot ),n-1,\cdot )}\right]\\ & & \;⪯\frac{1}{2{\left(\sigma_{k}^{-}\right)}^{3}}\left[\sum_{j^{\prime }=1}^{N}A_{kj^{\prime }}^{\left(\sigma \right)}P_{d}\left(\frac{m}{\tau_{kj^{\prime }}}\right)e^{-\frac{m}{\tau_{kj^{\prime }}}}+C_{k}^{\left(\sigma \right)}e^{-\frac{2m}{\tau_{L,k}}}\right]. \end{array}$$

Summarizing (60), (61), (62), (63)

varm[Xk(n−1,⋅)]⪯1σk−(∑j=1NAkj(det)Pd(mτkj)e−mτkj+Bk(det)e−mτL,k)+max[|θ−Vk−|,|θ−Vk+|]+max×1(2σk−)3[∑j′=1NAkj′(σ)Pd(mτkj′)e−mτkj′+Ck(σ)e−2mτL,k].

Therefore, we have:

$${\mathrm{var}}_{m}\left[X_{k}(n-1,\cdot )\right]⪯\sum_{j=1}^{N}A_{kj}^{\left(X\right)}P_{d}\left(\frac{m}{\tau_{kj}}\right)e^{-\frac{m}{\tau_{kj}}}+B_{k}^{\left(X\right)}e^{-\frac{m}{\tau_{L,k}}}+C_{k}^{\left(X\right)}e^{-\frac{2m}{\tau_{L,k}}},$$

for constants $A_{kj}^{\left(X\right)}$, $B_{k}^{\left(X\right)}$, $C_{k}^{\left(X\right)}$.

As a consequence,

$${\mathrm{var}}_{m}\left[p(n,\cdot )\right]⪯\frac{1}{\sqrt{2\pi }}\sum_{k=1}^{N}\left[\sum_{j=1}^{N}A_{kj}^{\left(X\right)}P_{d}\left(\frac{m}{\tau_{kj}}\right)e^{-\frac{m}{\tau_{kj}}}+B_{k}^{\left(X\right)}e^{-\frac{m}{\tau_{L,k}}}+C_{k}^{\left(X\right)}e^{-\frac{2m}{\tau_{L,k}}}\right].$$

Therefore, $\sum_{n\geq m^{\prime }}{\mathrm{var}}_{n-m^{\prime }}[p(n,\cdot )]$ is bounded from above by the series

12π∑k=1N[∑j=1NAkj(X)∑n≥m′Pd(n−m′τkj)e−n−m′τkj+Bk(X)∑n≥m′e−n−m′τL,k12π∑k=1N[+Ck(X)∑n≥m′e−2(n−m′)τL,k]=12π∑k=1N[∑j=1NAkj(X)∑l≥0Pd(lτkj)e−lτkj+Bk(X)∑l≥0e−lτL,k12π∑k=1N[+Ck(X)∑l≥0e−2lτL,k],

which converges, uniformly in $m^{\prime }$. As a consequence, in (52), v(p)<+∞ and we are done.

## Competing interests

The author declare that he has no competing interests.

## Footnotes

^1^We assume that all neurons have the same firing threshold. The notion of threshold is already an approximation which is not sharply defined in Hodgkin-Huxley [35] or Fitzhugh-Nagumo [40,41] models (more precisely it is not a constant but it depends on the dynamical variables [55]). Recent experiments [62-64] even suggest that there may be no real potential threshold.

^2^In [50], the authors use the following definition for the *nm* variation, which reads in our notations:

$${\mathrm{var}}_{m}^{\prime }\left[p(n,\cdot )\right]=\sup\left\{\left|p(n,\omega )-p\left(n,\omega^{\prime }\right)\right|;\omega ,\omega^{\prime }\in A_{-\infty }^{n},\omega_{m}^{n}=\omega_{m}^{n}\right\},\;m\leq n.$$

It differs therefore slightly from our definition (5), (38). The correspondence is ${\mathrm{var}}_{m}^{\prime }[p(n,\cdot )]={\mathrm{var}}_{n-m}[p(n,\cdot )]$. The definition of v(p) takes this correspondence into account.

^3^For D>0, this is $Z(\omega_{-D}^{-1})=\sum_{\omega (0)}e^{\phi^{\left(D\right)}(\omega_{-D}^{0})}$, ensuring that $\frac{e^{\phi^{\left(D\right)}(\omega_{-D}^{0})}}{Z(\omega_{-D}^{-1})}$ is a conditional probability $P[\omega (0)|\omega_{-D}^{-1}]$. Hence, it is not a constant but a function of the past $\omega_{-D}^{-1}$, in a similar way to statistical physics on lattices where the partition function depends on the boundary conditions. Only in the case D=0 (memory less) is this a constant.
